# Chiral Resolution of Spin‐Crossover Active Iron(II) [2x2] Grid Complexes

**DOI:** 10.1002/chem.202101432

**Published:** 2021-07-20

**Authors:** Nithin Suryadevara, Ansgar Pausch, Eufemio Moreno‐Pineda, Asato Mizuno, Jochen Bürck, Ananya Baksi, Tim Hochdörffer, Ivan Šalitroš, Anne S. Ulrich, Manfred M. Kappes, Volker Schünemann, Wim Klopper, Mario Ruben

**Affiliations:** ^1^ Institute of Nanotechnology (INT) Karlsruhe Institute of Technology (KIT) Hermann-von-Helmholtz-Platz 1 76344 Eggenstein-Leopoldshafen Germany; ^2^ Institute of Physical Chemistry (IPC) Karlsruhe Institute of Technology (KIT) Fritz-Haber-Weg 2 76131 Karlsruhe Germany; ^3^ Depto. de Química-Física, Escuela de Química Facultad de Ciencias Naturales, Exactas y Tecnología Universidad de Panamá 0824 Panamá Panamá; ^4^ Institute of Biological Interfaces (IBG-2) Karlsruhe Institute of Technology (KIT) Hermann-von-Helmholtz-Platz 1 76344 Eggenstein-Leopoldshafen Germany; ^5^ Fachbereich Physik Technische Universitat Kaiserslautern (TUK) Erwin-Schrödinger-Strasse 46 67663 Kaiserslautern Germany; ^6^ Department of Inorganic Chemistry Faculty of Chemical and Food Technology Slovak University of Technology in Bratislava Bratislava 81237 Slovakia; ^7^ Central European Institute of Technology Brno University of Technology Purkyňova 123 61200 Brno Czech Republic; ^8^ Department of Inorganic Chemistry, Faculty of Science Palacký University 17. listopadu 12 771 46 Olomouc Czech Republic; ^9^ Centre Européen de Sciences Quantiques (CESQ) Institut de Science et d'Ingénierie Supramoléculaires (ISIS) 8 allée Gaspard Monge, BP 70028 67083 Strasbourg Cedex France; ^10^ Institute for Quantum Materials and Technologies (IQMT) Karlsruhe Institute of Technology (KIT) Hermann-von-Helmholtz-Platz 1 76344 Eggenstein-Leopoldshafen Germany

**Keywords:** chirality, grid complexes, ion mobility, Mössbauer spectroscopy, spin-crossover

## Abstract

Chiral magnetic materials are proposed for applications in second‐order non‐linear optics, magneto‐chiral dichroism, among others. Recently, we have reported a set of tetra‐nuclear Fe(II) grid complex conformers with general formula C/S‐[Fe_4_L_4_]^8+^ (L: 2,6‐bis(6‐(pyrazol‐1‐yl)pyridin‐2‐yl)‐1,5‐dihydrobenzo[1,2‐d : 4,5‐d′]diimidazole). In the grid complexes, isomerism emerges from tautomerism and conformational isomerism of the ligand L, and the S‐type grid complex is chiral, which originates from different non‐centrosymmetric spatial organization of the *trans* type ligand around the Fe(II) center. However, the selective preparation of an enantiomerically pure grid complex in a controlled manner is difficult due to spontaneous self‐assembly. To achieve the pre‐synthesis programmable resolution of Fe(II) grid complexes, we designed and synthesized two novel intrinsically chiral ligands by appending chiral moieties to the parent ligand. The complexation of these chiral ligands with Fe(II) salt resulted in the formation of enantiomerically pure Fe(II) grid complexes, as unambiguously elucidated by CD and XRD studies. The enantiomeric complexes exhibited similar gradual and half‐complete thermal and photo‐induced SCO characteristics. The good agreement between the experimentally obtained and calculated CD spectra further supports the enantiomeric purity of the complexes and even the magnetic studies. The chiral resolution of Fe(II)‐ [2×2] grid complexes reported in this study, for the first time, might enable the fabrication of magneto‐chiral molecular devices.

## Graphical abstract

**The** ***trans*** **‐type ligand** wrapping around the metal centers in an inter‐woven style makes the complex to be in chiral. This separation of **Λ** and **Δ** forms of spin‐crossover isomers was successfully achieved by appending the chiral moiety onto the parent ligand. The presence of spin‐crossover and chirality makes these multifunctional complexes intriguing for the study of magneto‐optical effects.

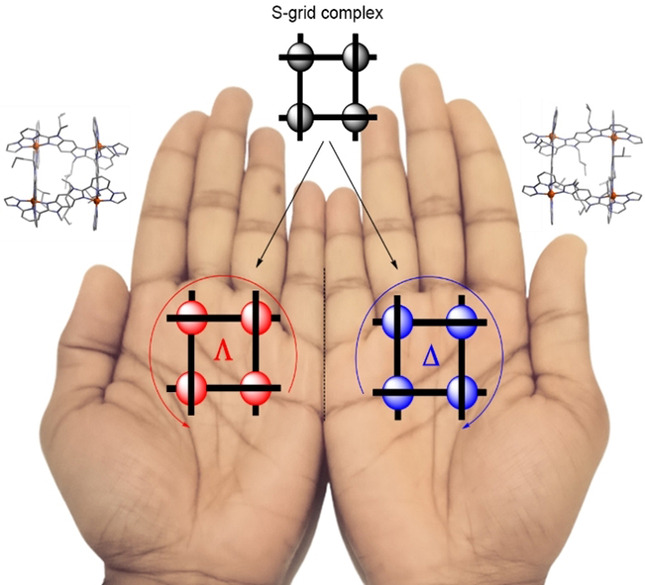

## Introduction

Chirality is one of the most central concepts in molecular sciences. Chiral materials can be found in a wide range of fields such as biology, medicine,^[1]^ magnetism,[^2^, ^3^] spintronics,[^4^, ^5^] and non‐linear optics.[^6^, ^7^] Since the observation of the magneto‐chiral dichroism (MChD) effect in chiral paramagnetic materials,^[8]^ the investigation of chiral magnets has become an active research topic, and multifunctional materials combining magnetism and chirality have been proposed for applications. In 1999, Ron Naaman and co‐workers observed that the interaction of electrons with chiral molecules is spin specific, leading to spin‐filtering termed as chiral induced spin selectivity (CISS) effect.^[9]^ This spin‐filtering ability of chiral molecules has opened up the possibility of using chiral molecules in spintronic devices.^[4]^

Spin‐crossover (SCO) complexes are the well‐known class of switchable molecular materials capable of undergoing a reversible transition between low‐spin (LS) and high‐spin (HS) states as a function of temperature, pressure, light irradiation or exposure to an electric field.[^10^, ^11^, ^12^, ^13^] The hysteretic nature of SCO associated with some of the SCO complexes makes them suitable to fabricate molecular electronic/spintronic architectures.[^10^, ^13^, ^14^, ^15^, ^16^, ^17^, ^18^] Introducing chirality into SCO complexes is a promising strategy to obtain novel magneto‐optical materials.^[19]^ Such chiral materials could be optically triggered/addressed, making them suitable for the fabrication of molecular‐and photonic devices.^[20]^ It has been shown that chiral SCO materials could facilitate non‐destructive readout of storage media, using an optical rotation measurement.^[21]^ In this context, Ohkoshi et al. reported spin‐crossover‐induced second‐harmonic generation (SHG), light‐reversible SCO long‐range magnetic ordering, and photo‐reversible switching of the magnetic SHG effect based on a chiral 3D‐cyano‐bridged Fe−Nb bimetallic assembly.^[19]^

**Figure 1:**
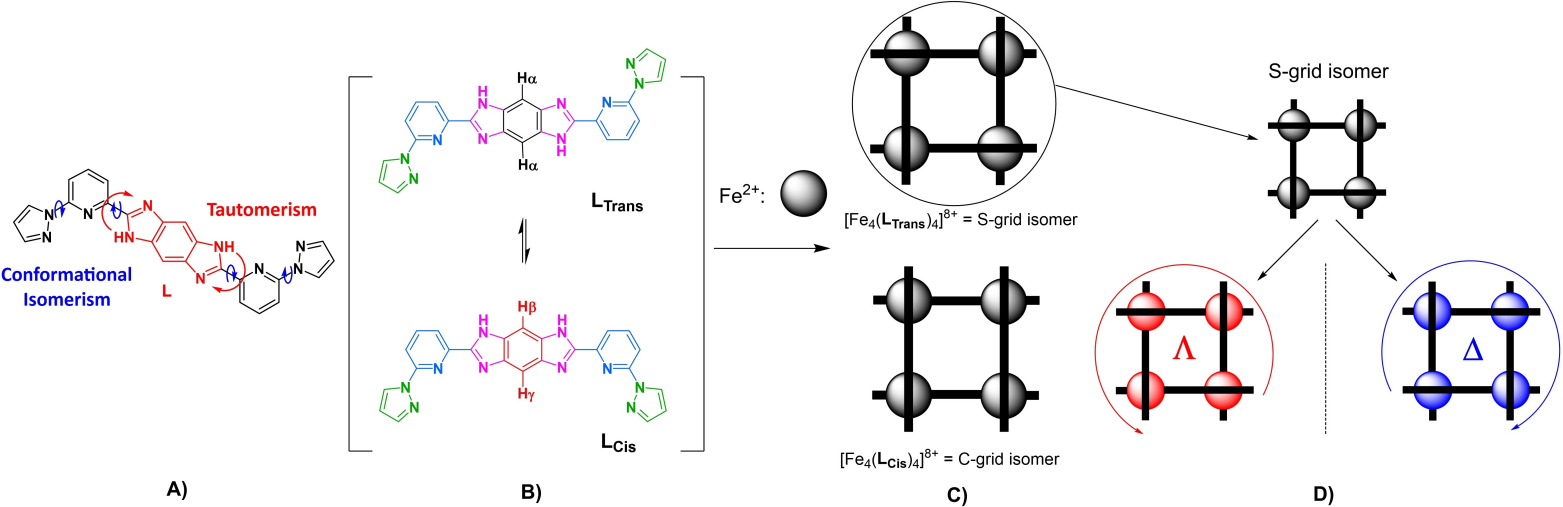
A) The conformational isomerism and tautomerism in ligand **L**. B) **L_Trans_** and **L_Cis_** conformers of **L** in solution. C) Divergent coordination chemistry of ligand **L** (black bar) on coordination with Fe^2+^ metal ions (grey balls). D) **Λ** and **Δ**‐isomers of the S‐grid complex.

In the family of poly‐nuclear complexes, metallo‐grid complexes stand out in the field of magneto‐chemistry because of their tunable magnetic properties, which can be modulated by varying the ligands and metal centers.^[22]^ Ever since the first report on SCO active grid complexes by Lehn and others, valiant attempts have been directed towards the realization of SCO active grid complexes. Efforts have been made to elucidate the structure‐SCO property relationship and the utility of grid complexes for device fabrication.^[23]^ In 2016, our group has reported a parallel synthesis of Fe(II) grid complex tauto‐conformers based on a homoditopic ligand, which consists of two tridentate 2‐(1*H*‐imidazol‐2‐yl)‐6‐(pyrazol‐1‐yl)pyridine units interlinked via a central benzo[1,2‐*d* : 4,5‐*d′*]diimidazole bridge (Figure 1a).^[24]^ In the same report, the two conformers (S‐form and C‐form), which were caused by tautomerism and conformational isomerism in the ligand (Figure 1b), were separated by fractional crystallization. They showed contrasting magnetic behavior; the S‐form showed gradual SCO, whereas the C‐form is completely trapped in the HS state.^[24]^

Chirality can be observed in such grid complexes composed of ligands in an interwoven arrangement. In the S‐type grid complex, the four ligands wrap around the four metal centers in a helically interwoven fashion, resulting in the formation of chiral grid complexes, i.e, in the form of enantiomerically pure Δ and Λ forms (Figure 1d). Thus, the S‐type complexes are of interest for applications due to their intrinsically chiral nature. Moreover, the different spatial organization of the ligand around Fe(II) centers and consequent molecular packing in the crystal lattice could lead to enantiomer‐specific SCO characteristics. To harness magneto‐chiral applications, the chiral resolution of S‐grid complexes is necessary and is a challenging task. The majority of the chiral SCO complexes reported[^21^, ^25^] till today have been obtained by spontaneous resolution, i. e., generation of Δ and Λ crystals simultaneously in the mixture instead of enantiomerically pure complexes. Moreover, it is also challenging to prepare enantiomerically pure complexes in large quantities.

So far, different strategies were developed to solve the problem of isomeric deconvolution, such as using chiral counter anions,^[26]^ or by introducing chirality in the ligand.^[27]^ To the best of our knowledge, the resolution and the studies of two enantiomers of Fe(II) SCO grid complexes have not been reported yet. Moreover, the chiral resolution must be achieved to investigate the relationship between SCO parameters and inbuilt chiral information. In this study, we report on the large scale separation of the two enantiomers of Fe(II) S‐grids achieved by modifying the parent ligand with chiral moieties.

## Results and Discussion

### Synthesis of ligands and complexes

**Scheme 1:**
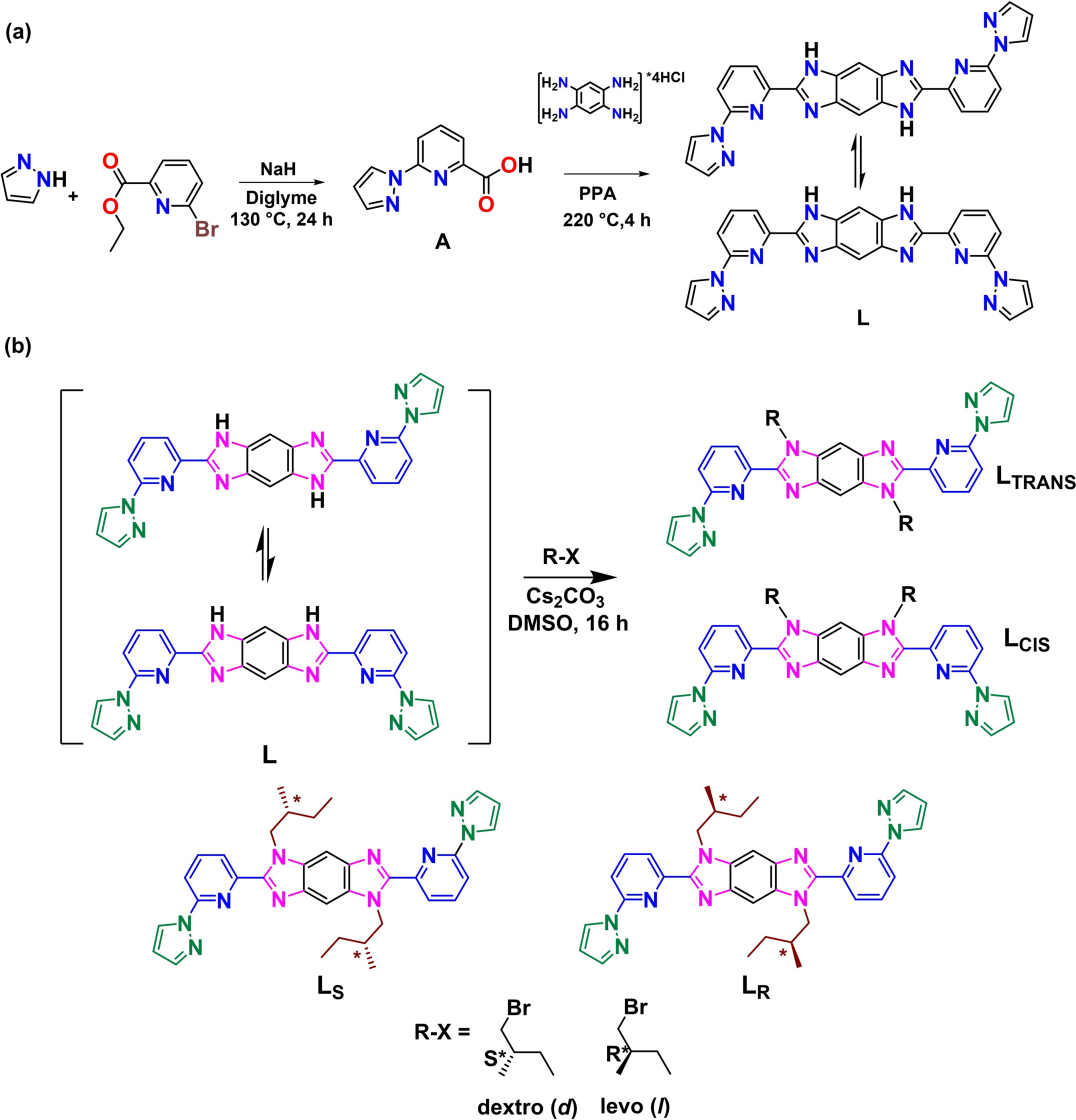
Schematic representation of the synthesis of (a) ligand **L**, and (b) chiral ligands **L_S_** and **L_R_**.

The parent ligand (**L**) was synthesized by the condensation reaction between the 6‐(1*H*‐pyrazol‐1‐yl)picolinic acid (**A**) and 1,2,4,5‐ benzene‐tetraamine in polyphosphoric acid (PPA) (Scheme 1a and Figure SI‐1). As reported,^[24]^ the ^1^H NMR spectroscopic investigations showed the presence of **L_Trans_** and **L_Cis_** tauto‐conformers in a 1 : 1 molar ratio. This tautomerization in the ligand leads to different coordination modes of the ligand and eventually to the formation of different conformers of metal complexes (Figure 1c). By introducing chemical substitution at the secondary amine group on the parent ligand **L** to form the tertiary amine, tautomerization can be blocked.^[28]^ Due to the difference in structural conformations, the cis‐ and trans‐type of ligands show different polarities, and this facilitates the separation of the cis‐ and trans‐ ligands by traditional column chromatography.

The treatment of parent ligand **L** with chiral (R)‐ and (S)‐alkyl bromides in DMSO in the presence of Cs_2_CO_3_ as a base produced two sets of enantiomerically pure cis (R‐cis and S‐cis) and trans (R‐trans and S‐trans) isomers. Thus, tautomerism is prevented on the ligand **L** (Scheme 1b). ^1^H NMR measurements confirmed the cis‐ and trans‐ ligands, as they show different shifts for protons on benzene rings (see Figure SI‐3 and Figure SI‐4). Chiral HPLC was used to confirm the enantiopurity of the formed trans ligands and was found to be 94 % and 87 % pure with along with some formation of meso product (see Figure SI‐10).

**Figure 2:**
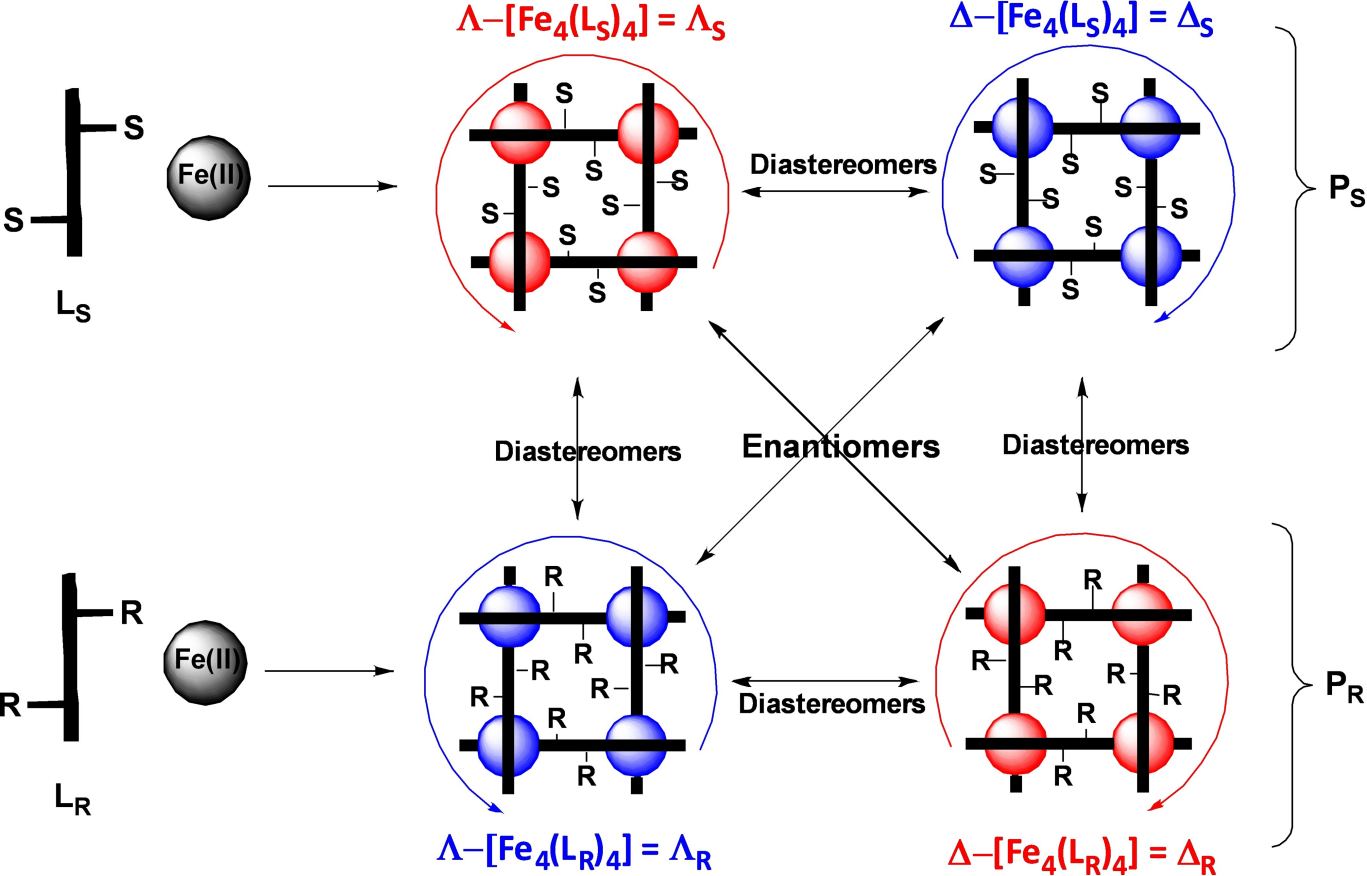
Scheme for the synthesis of isomeric grid complexes representing the pairs of diastereomers and enantiomers.

Since our interest is on the non‐centrosymmetric S‐type grid complex, we primarily focus here on trans type ligand. Based on the specific rotation of the chiral moiety affixed, we denoted the new trans ligands by **L_S_** and **L_R_** (Scheme 1). The reaction of the newly formed trans ligands (**L_S_** and **L_R_**) with equimolar amounts of Fe(CF_3_SO_3_)_2_ in acetonitrile yielded two sets of diastereomeric products **P_S_** ((**Λ**)‐[Fe_4_(L_S_)_4_] and (**Δ**)‐[Fe_4_(L_S_)_4_]) and **P_R_** ((**Λ**)‐[Fe_4_(L_R_)_4_] and (**Δ**)‐[Fe_4_(L_R_)_4_]) (Figure 2). Several strategies, such as using chiral solvents and chiral counter anions (for crystallization), and recrystallization with different solvents, were adopted to produce pure diastereomers. Among these, repeated recrystallization of the reaction products from acetonitrile helped the separation of pure diastereomers. The ion mobility mass spectrometry (IMMS)was used to confirm the presence of diastereomers. Further, the purity of the complexes was also elucidated by performing XRD and CD spectroscopic studies.

### Electrospray ionization (ESI) and ion mobility mass spectrometric (IMMS) studies

Electrospray ionization‐mass spectrometry (ESI‐MS) was employed for the identification of the as‐synthesized, purified, and crystallized samples. Details of the MS method are given in the Supporting Information. Briefly, about 10 μg/mL concentrated solution of each sample in acetonitrile was electro‐sprayed. The doubly charged ion peak was found in positive ion mode at m/z 1728 with a few fragments (Figure SI‐5).

**Figure 3:**
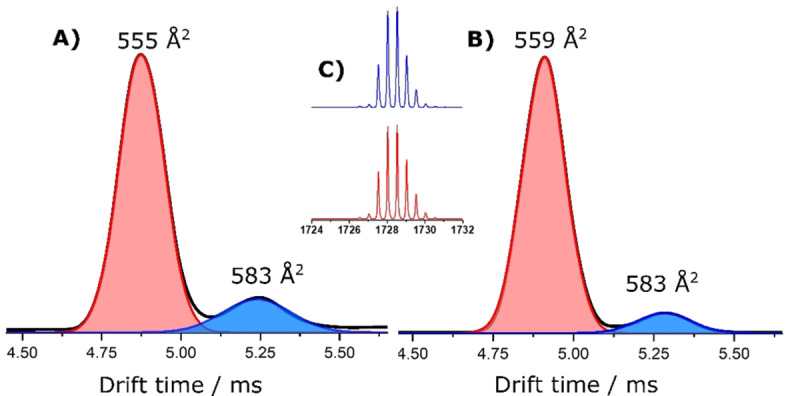
IMMS data of crude samples A) **P_S_** and B) **P_R_** showing the presence of two isomers (possibly two diastereomers) in both the samples. The mass spectrum extracted from the two peaks of **P_S_** is shown in inset C.

To understand the structural details and to find the presence of diastereomers in the samples, ion mobility mass spectrometry (IMMS) experiments were performed. Details of the IMMS methods are given in the Supporting Information. Extracted ion mobilograms for the peak m/z 1728 from **P_S_** and **P_R_** are shown in Figure 3a and 3b, respectively. The obtained ion mobilogram of each **P_S_** and **P_R_** for the peak at 1728 m/z showed two peaks, possibly indicating the presence of two diastereomers and their plausible assignments were given in Table 1. The peak with highest intensity **P**_***S***_ appeared at 4.86 ms while the next peak was found at 5.26 ms. From the area under the peaks, the abundance of the two isomers was calculated as 73 % and 27 %, respectively (Table 1). Collision cross‐section (CCS) of the ions was calculated using polyalanine dication as a calibrant. The CCS of **P_S_1** and **P_S_2** were 555 Å^2^ and 583 Å^2^, respectively. The respective mass spectrum extracted from each of the peaks is shown in the inset **C**. While the arrival time difference between the two diastereomers was 0.4 ms for **P_S_**, it was 0.34 ms for **P_R_** (Figure 3). The experimental CCS values for diastereomers from the other crude product **P_R_1** and **P_R_2** were 559 Å^2^ (drift time (dt): 4.92 ms) and 583 Å^2^ (dt: 5.26 ms), respectively. So this study shows the possibility of the presence of two diastereomers and the major products of both crude samples show approximately the same CSS values, which is anticipated for enantiomers.

**Table 1:** CCS and arrival times of different isomers formed.

Peak	Arrival Time [ms]	CCS [Å^2^]	Area [%]
P_S_1 (**Λ_S_**)	4.86	555	73
P_S_2 (**Δ_S_**)	5.26	583	27
P_R_1 (**Δ_R_**)	4.92	559	81
P_R_2 (**Λ_R_**)	5.26	583	19

### Single crystal X‐ray diffraction analysis

**Figure 4:**
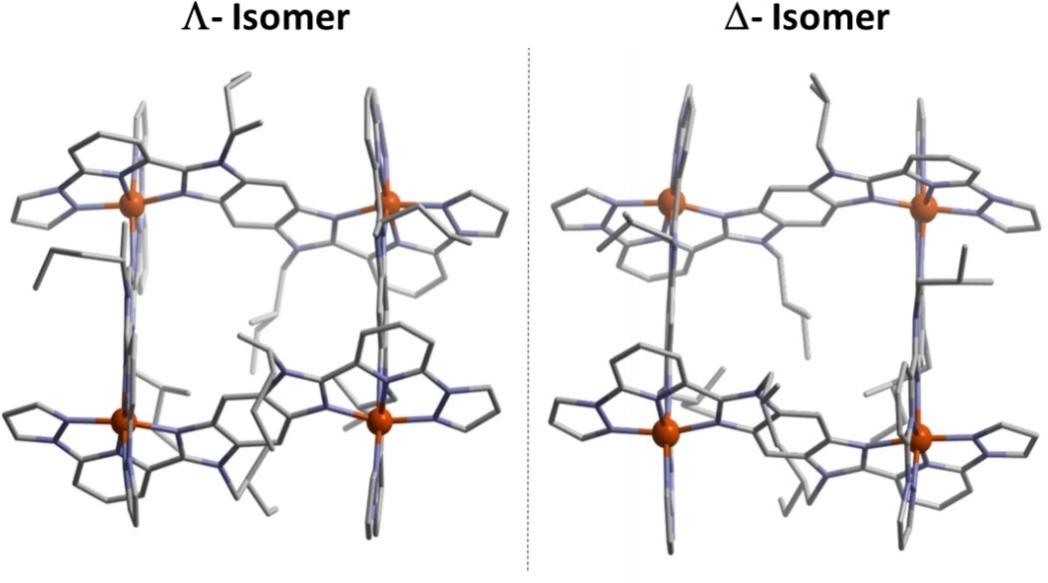
The single crystal X‐ray diffraction structures of enantiomeric Fe(II) grid complexes **Λ_S_** and **Δ_R_** (H atoms, counter anions, and solvent molecules were omitted for clarity).

To study their stereochemistry in detail, the crystal structures of the above‐synthesized complexes were obtained. The crystalline forms of grids **P_S_** and **P_R_** were obtained by slow vapor diffusion of THF into the solutions of the complexes in acetonitrile. The single‐crystal X‐ray investigations of the crystals carried out at 180 K revealed the expected [2×2] grid type molecular structures. Both isomers crystallized in a non‐centrosymmetric *I*4 space group, and they are isostructural and isomorphous to each other. Depending on the orientation of ligands around the Fe(II) centers, the configuration of crystals from the **P_S_** complex is **Λ_S_** and similarly **Δ_R_** from the **P_R_** complex, while both being enantiomers to each other (Figure 4). All the ligands and central atoms in the structure are crystallographically equivalent with fourfold symmetry. Each unit cell has one grid complex, with the asymmetric unit consists of the Fe**L_S_**/Fe**L_R_** moiety and one triflate anion. Although all the anions could not be located, elemental analysis confirmed the molecular formula of [Fe^(II)^ **L_S_**]_4_(Tf)_8_ (see Supporting Information). The four Fe(II) ions are located at the corners of the square with an adjacent Fe−Fe distance of 8.978(5) Å, whereas diagonal Fe ions are separated by 12.696(4) Å. Each Fe(II) ion was coordinated by six nitrogen atoms from nearly perpendicularly oriented ligands with a dihedral angle of 87.86°. At 180 K, the average Fe−N bond lengths of the **Λ_S_** and **Δ_R_** complexes are 1.941(2) Å and 1.951(2) Å, respectively, which corresponds to the LS state.^[29]^ The spin state of Fe(II)‐bis(pyrazol‐1‐yl)pyridine complexes can be confirmed from angular components, for example, the N_pyrazole_−Fe−N_pyrazole_ angles (*ϕ*), which is about 160° for LS and 145° for HS and the distortion indices *Σ* which is ca. 90° for LS and 160° for HS.[^30^, ^31^, ^32^] From the crystal structures, the calculated average values of N_pyrazole_−Fe−N_imidazole_ angles (*ϕ*) are 159.4(4)° and 160.7(6)° for **Λ_S_** and **Δ_R_**, respectively, confirming the LS for both structures. The bond lengths and the angular parameters are collected in Table SI‐3 and further details are given in the Supporting Information.

### Circular dichroism studies and its comparison with time‐dependent density functional theory (TDDFT) calculations

Circular dichroism (CD) is a well‐established technique for studying chiral molecules, that measures the difference in absorbance of the left‐ and right‐handed circularly polarized light by chiral compounds as a function of wavelength. The line shape of the spectrum obtained is characteristic for the absolute configuration of the synthesized complex. Thus, to further analyze the stereochemistry of the complexes, the CD spectra of the complexes were studied in the solution‐state.[^33^, ^34^] The crude sample from each reaction, used for IMMS, was dissolved in acetonitrile and was subjected to CD measurements. The experiments showed weak signals, confirming a small diastereomeric excess in the crude samples (see Figure SI‐6), which is in agreement with the IMMS studies. However, this is in contrast to the XRD measurements, where the prepared samples were enantiopure. To understand this problem, we measured the CD for several single crystals selected manually out of the crystal mixture by dissolving them in acetonitrile. Some of the crystals showed pronounced peaks with Δ*ϵ* values of about +/−300 L mol^−1^ cm^−1^ at 383 nm, whereas some other crystals showed a major difference in terms of Δ*ϵ* signal, which is lesser than 300 L mol^−1^ cm^−1^ (see Figure SI‐6). From this observation, it was clear that, on crystallization, only a few crystals formed were pure diastereomers, whereas the majority were not. Therefore, to achieve pure diastereomers in each case, we performed several recrystallizations, and over every recrystallization cycle, we found an improvement of signals for the bulk sample (see Figure SI‐7). After four recrystallizations, we achieved Δ*ϵ* intensities of the complexes, which were the same as that of the corresponding single crystal, and this remained the same even on further recrystallizations. Thus, several steps of recrystallizations have provided a new strategy to produce pure diastereomers in each case.

**Figure 5:**
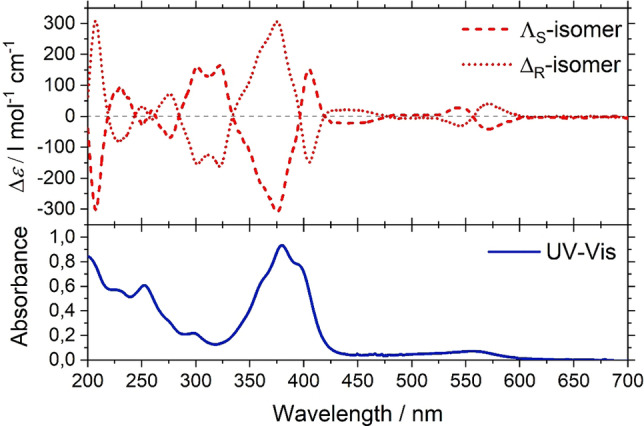
CD and UV‐Vis of **Λ_S_** and **Δ_R_**‐Fe(II) grid complexes measured in acetonitrile as solvent.

In Figure 5 the CD and UV‐Vis spectra of the fully resolved enantiomers recorded in acetonitrile solution at 25 °C are shown. The two intense bands at 270 nm and 370 nm are due to ligand‐centered (LC) π→π* transitions.^[35]^ The band at 550 nm corresponds to the metal‐to‐ligand‐charge‐transfer (MLCT) transition, which is observed in LS complexes.^[35]^ All these bands showed strong CD activities for the pure **Λ_S_** and **Δ_R_** enantiomers. Moreover, the CD signals were found to be exact mirror images with opposite sign of the bands. The most prominent feature in the spectra is the bisignate Cotton effect due to exciton coupling around 370 nm, which has a negative band for the **Λ_S_** isomer and a positive band for the **Δ_R_** isomer.

**Figure 6:**
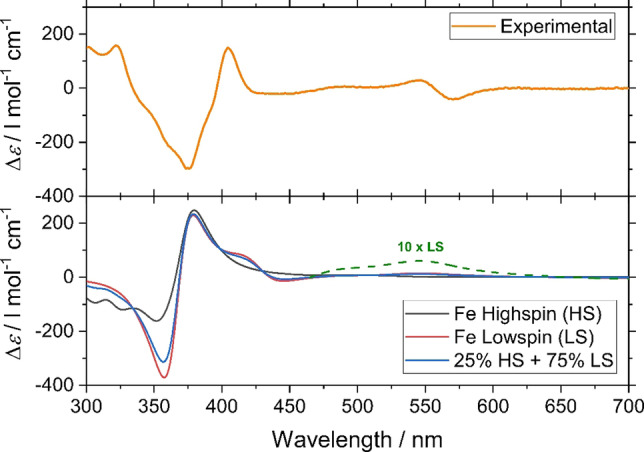
Comparison of experimental CD (top) and simulated CD spectra (bottom) of all **Λ_S_**‐Fe(II) grid complex.

Time‐dependent density functional theory (TDDFT) has become a standard tool in the evaluation of excited‐state properties.[^36^, ^37^, ^38^, ^39^] For the simulation of CD spectra, in particular, TDDFT has been proven to be an accurate and reliable method capable of handling molecules with up to hundreds of atoms.[^40^, ^41^, ^42^, ^43^, ^44^] Calculations were performed on the low‐spin and high‐spin complexes and before calculating their respective TDDFT spectra, the geometries of the complexes were optimized using Turbomole.[^45^, ^46^, ^47^] The different spectra are shown in Figure 6.

**Figure 7:**
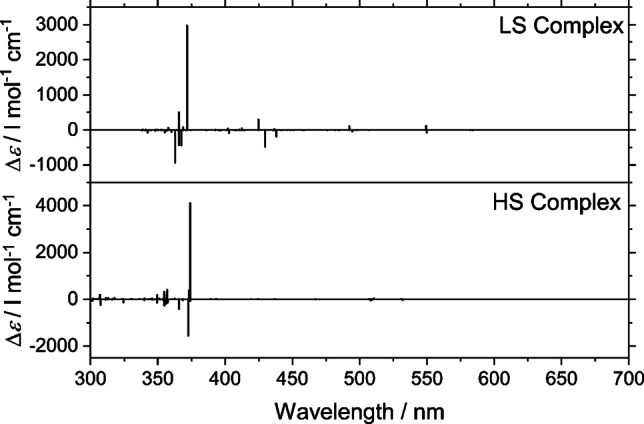
Simulated line spectra of Low‐spin (top) & High‐spin (bottom) **Λ_S_**‐Fe(II) grid complex.

From the magnetic measurements, it was inferred that around 25 % and 75 % of the molecules are in the HS and LS states, respectively, at RT (see below). To mimic the RT situation in the calculations, we included a weighted average of the LS and HS spectra, which was compared to the experimental one. Overall, the calculated and the experimental spectra showed good agreement both qualitatively and quantitatively. The MLCT bands at around 550 nm, however, are significantly smaller in the calculated spectrum. This could be explained by the fact that hybrid functionals are known to underestimate charge‐transfer (CT) excitations. We additionally performed calculations using the CAM‐B3LYP functional,^[48]^ which was designed to capture the effects of CT excitations better. The results of the CAM‐B3LYP calculations did not systematically improve upon the PBE0 calculations: the intensity of the MLCT bands was even smaller than the intensities obtained from the PBE0 functional. One feature which is visible in the simulated spectra is the presence of the MLCT bands in the spectrum of the LS complex, whereas they are absent in the spectrum of the HS complex, as shown in Figure 7.

This is in full agreement with the experimental results, and also with the magnetic measurements. The presence of the MLCT bands, therefore, indicates the presence of the LS iron(ii) centers associated with the complex. The comparable nature of the computed CD spectra obtained from TDDFT calculations has validated the enantiomeric purity of the obtained complex.

### Temperature and light‐induced spin‐state switching characteristics of the [2×2] grid complexes

**Figure 8:**
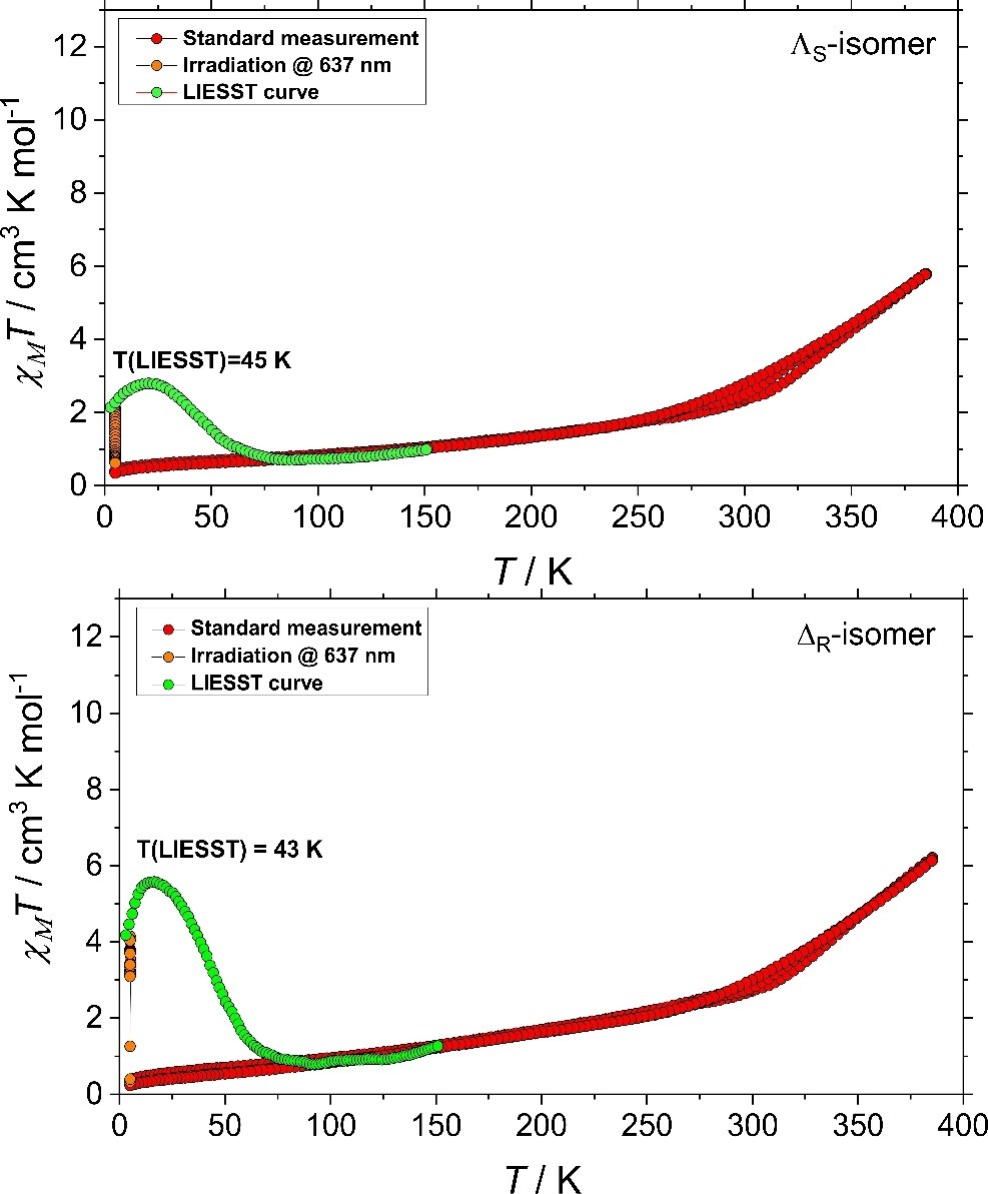
*χ*_Μ_ *T* vs. *T* plots and photo‐magnetic characteristics of **Λ_S_** and **Δ_R_**‐complexes.

The studies of thermal and photo‐induced SCO behavior were carried out by variable temperature magnetic investigation in the thermal range 5–385 K. The *χ*_Μ_ *Τ* vs. *T* dependencies indicate the presence of gradual and high‐temperature SCO behavior in both heating and cooling modes (Figure 8). At room temperature (≈300 K), the *χ*_Μ_ *Τ* value is close to ca. 2.84 (for **Λ_S_**‐isomer) and 2.92 (for **Δ_R_**‐isomer) cm^3^ K mol^−1^, which corresponds to ca. 25 % of the complexes is in the HS state. The values of *χ*_Μ_ *Τ* product at the maximal temperature of measurement are ca. 5.88 (**Λ_S_**‐isomer, at 385 K) and 6.14 (**Δ_R_**‐isomer, at 385 K) cm^3^ K mol^−1^ and suggest approximately 50 % of Fe(II) metal centers in HS state. Below 180 K, *χΤ* value is close to 1.00 cm^3^ K mol^−1^, which shows most of the metal centers are in diamagnetic LS state, corroborating well with the spin‐state information obtained from the crystallographic studies. The measurements for both complexes were performed in the several heating and cooling modes. The tiny differences between the curves in corresponding measurement cycles are tentatively attributed to the partial loss of lattice solvents or adsorbed moisture.

In order to study and compare the light‐induced SCO behavior in the enantiomeric grid complexes, light‐induced excited spin‐state trapping (LIESST) measurements, both LS (at 5 K) enantiomeric samples were irradiated with red light (*λ* =637 nm, power=10 mW/cm^2^) in the dark for ca. 2 h. A significant increase of magnetization was observed under the irradiation, which is an indisputable proof for the formation of photo‐excited metastable HS state. The progress of the magnetization was followed with time, and when saturation was reached, the light was switched off and temperature‐dependent magnetization was recorded in the dark. Both isomers exhibit similar photo‐magnetic properties with partial LS→HS photo‐conversion (≈23 % for **Λ_S_**‐isomer and ≈46 % for **Δ_R_**‐isomer) and gradual thermal relaxation upon the slow heating. The various amounts of the photo‐excited metastable HS state for **Λ_S_** and **Δ_R_** might be attributed to the different penetration of light through the prepared samples for the irradiation experiments.^[49]^ On the other hand, *T*(LIESST) temperatures are very similar to each other and correspond to high *T*_1/2_ temperature of thermal SCO, which agrees with the “inverse energy gap law,” which states *T*(LIESST) is inversely proportional to *T*_1/2_.[^50^, ^51^]

### Mössbauer spectroscopy

^57^Fe Mössbauer spectroscopy is also another valuable tool for accessing the spin and charge state of the Fe atoms.^[52]^ So, in order to study the spin states at various temperatures, Mössbauer spectra were measured for the ^57^Fe enriched **Λ_S_** and **Δ_R_** enantiomeric complexes at 77 K and 300 K temperature. The parameters obtained are compiled in the Table 2.

**Table 2:** The isomer shifts (*δ*) and quadrupole splittings (Δ*E*_Q_) of enantiomeric complexes at 77 K and 300 K.

*T*/K	Complex	*δ* [mm/s]	Δ*E*_Q_ [mm/s]	Area [%]
77	**Λ_S_**‐isomer (LS)	0.38(1)	0.49(2)	91(1)
	**Λ_S_**‐isomer (HS)	1.10(1)	2.54(2)	9(1)
77	**Δ_R_**‐isomer (LS)	0.38(1)	0.49(2)	93(1)
	**Δ_R_**‐isomer (HS)	1.10(1)	2.54(2)	7(1)
300	**Λ_S_**‐isomer (LS)	0.32(1)	0.47(2)	78(1)
	**Λ_S_**‐isomer (HS)	0.78(1)	1.80(2)	22(1)
300	**Δ_R_**‐isomer (LS)	0.30(1)	0.48(2)	83(1)
	**Δ_R_**‐isomer (HS)	0.78(1)	1.82(2)	17(1)

**Figure 9:**
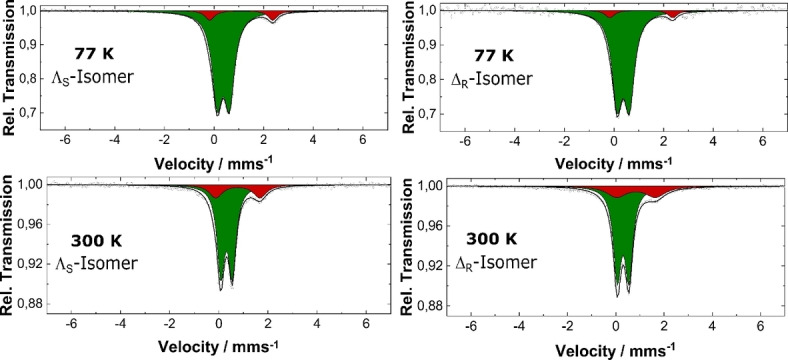
Mössbauer spectra for ^57^Fe enriched **Λ_S_** and **Δ_R_** complexes at 77 K and 300 K, green represents LS, and red represents HS.

The spectra (Figure 9) feature two doublets at 77 K for both isomers (for **Λ_S_** and **Δ_R_**) with isomer shifts (*δ*) of 0.38 and 1.10 mm/s. The quadrupole splittings (Δ*E*_Q_) obtained are, Δ*E*_Q_=0.49 mm/s which corresponds to LS−Fe(II) ions, and Δ*E*_Q_=2.54 corresponding to HS−Fe(II).^[53]^ The area under the peaks confirms around ca. 8 % HS content at 77 K. This is in good agreement with the magnetic measurements, which showed non‐zero *χΤ* values. On the other hand, the spectra obtained at RT features well‐separated quadrupole doublets for both isomers, confirming the two spin states of the iron ions. The figure shows the green doublet with *δ*=0.32 mm/s, (and Δ*E*_Q_=0.47 mm/s for **Λ_S_**) and *δ*=0.30 mm/s (and Δ*E*_Q_=0.48 mm/s for **Δ_R_**) corresponding to the LS content in the samples. Whereas, the red doublet at δ=0.78 mm/s, (with Δ*E*_Q_=1.80 mm/s for **Λ_S_**) and *δ*=0.78 mm/s (Δ*E*_Q_=1.82 mm/s for **Δ_R_**) corresponds to HS−Fe(II) (Figure 9). The area under the peaks confirms around ca. 20 % HS content at RT.

Magnetic measurements of these resolved enantiomers **Λ_S_** and **Δ_R_** shows the presence of ∼25 % HS‐state, which is corroborating with Mössbauer measurements at 300 K. On the other hand, at 180 K, the crystal structure shows LS structure which is consistent with the magnetic data. The presence of LS state is also proved from CD data which shows MLCT band around 550 nm. These complexes resolved and discussed in this report stand out as new example for chiral SCO complexes where helical chirality is intrinsic in the coordination environment. Previously, Bark and coworkers reported on successful chiral resolution of Fe(II) grid complexes using a chiralized version of the ligand, where the chiral moiety blocks the formation of another diastereomer with steric hindrance.[^27^, ^54^] However, in the present case, the resolution was not achieved in a single step, which could be due to the weak interaction of the chiral unit with the neighboring ligands, resulting in the formation of a product with small diastereomeric excess.

Remarkably, the critical step that helped us in the purification of diastereomers was crystallization steps. Chiral self‐sorting effects could have possibly helped during the self‐assembly process. Chiral self‐sorting is a phenomenon where racemic components are spontaneously sorted into homo‐ or hetero‐chiral molecular assemblies through chiral discrimination between the components.[^55^, ^56^, ^57^] The presence of some pure diastereomeric crystals from the first crystallization step could have helped by taking on the role of the seed crystal and undergo preferential crystallization.^[58]^ This helped to improve the diastereoselectivity, as evidenced by CD measurements. In general, preferential crystallization requires the addition of an external seed crystal. However, in the case of here, the in situ generated crystal has helped as a seed crystal for successive stages of crystallization to produce a pure diastereomeric product.

## Conclusion

In conclusion, we were able to separately synthesize the first set of enantiomers of SCO [2×2] grid complexes by tailoring the 2,6‐bis(6‐(pyrazol‐1‐yl)pyridin‐2‐yl)‐1,5‐dihydrobenzo[1,2‐d : 4,5‐d′]diimidazole ligand system with chiral centers. IMMS experiments confirmed the formation of diastereomers during the reaction. The stereochemistry of the complexes was confirmed by XRD and CD measurements. Magnetic measurements have shown the gradual temperature‐induced SCO behavior in these complexes, and Mössbauer measurements confirmed the spin states of the complexes. The complexes were LIESST active at low temperatures. Moreover, TDDFT calculations provided additional evidence to both structural and magnetic studies. Such multifunctional architectures, possessing chirality and SCO behavior, are of interest for future applications since they can offer magneto‐chiral effects. Thus, it also represents a promising model for the development of future memory molecular devices.^[59]^ Furthermore, this study has opened the track for preparing other functional molecules by tuning ligand and metal center – such that one could design hetero‐metallic grid like architectures with other functional properties.

## Experimental Section

**General**: Purchased chemicals and solvents were obtained from commercial suppliers and were used without any further purification. The chiral reagent – (S)‐(+)‐1‐bromo‐2‐methylbutane was obtained as 99 % purity from Sigma‐Aldrich. The other chiral reagent ‐ (R)‐(−)‐1‐bromo‐2‐methylbutane was obtained as ≥95 % purity from Angene Chemicals, China. All the reactions were performed under argon(Ar) atmosphere. ^1^H and ^13^C NMR, correlation measurements were performed using a Bruker Ultrashield plus‐500 spectrometer with solvent proton as an internal standard. FTIR spectra were measured using KBr pellets (Magna FTIR 750, Nicolet) in the region of 4000–400 cm^−1^. Mass spectrometric data were obtained with MicroTOF‐Q II Brucker for ESI‐TOF.

Detailed experimental procedures, instrumentation and analytical data are provided in the Supporting Information. Deposition Numbers 2064448 (for **Λ**_**S**_) and 2064449 (for **Δ**_**R**_) contain the supplementary crystallographic data for this paper. These data are provided free of charge by the joint Cambridge Crystallographic Data Centre and Fachinformationszentrum Karlsruhe Access Structures service www.ccdc.cam.ac.uk/structures.

## Conflict of interest

The authors declare no conflict of interest.

## Supporting information

As a service to our authors and readers, this journal provides supporting information supplied by the authors. Such materials are peer reviewed and may be re‐organized for online delivery, but are not copy‐edited or typeset. Technical support issues arising from supporting information (other than missing files) should be addressed to the authors.

Supporting Information
