# Supplementary material for: Chiral Resolution of Spin‐Crossover Active Iron(II) [2x2] Grid Complexes
Source: Chemistry. 2021 Jul 20;27(61):15172–80. doi: 10.1002/chem.202101432 (PMC8597157; doi:10.1002/chem.202101432)
Supplement: Supplementary file 1 — Supporting Information [file CHEM-27-15172-s001.pdf]

Chemistry–A European Journal

Supporting Information

Chiral Resolution of Spin-Crossover Active Iron(II) [2x2] Grid Complexes

Chemistry–A European Journal

Supporting Information

Chiral Resolution of Spin-Crossover Active Iron(II) [2x2] Grid Complexes

Table of content:

1. Ligand synthesis

2. Synthesis of [2x2]-iron(II) grid complexes

3. ESI-MS and IMMS

4. Circular dichroism (CD) of Grid complexes

5. Deconvolution of diastereomeric grid complexes

6. X-ray diffraction analysis

7. TDDFT calculations

8. Magnetic measurements

9. Mossbauer measurements

10. Differences in ECD spectra between Λ_S and Λ_R complexes

11. References

1. Ligand Synthesis:

Scheme 1:

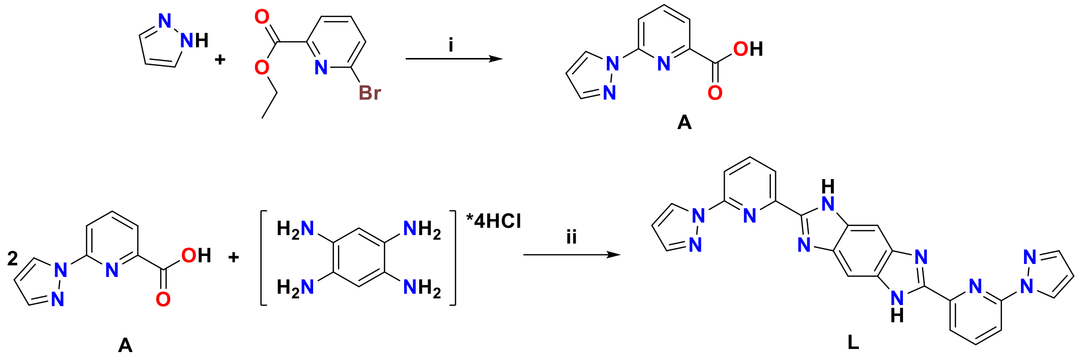

Figure SI-1: *Scheme for the synthesis of ligand* ***L***

Scheme 2:

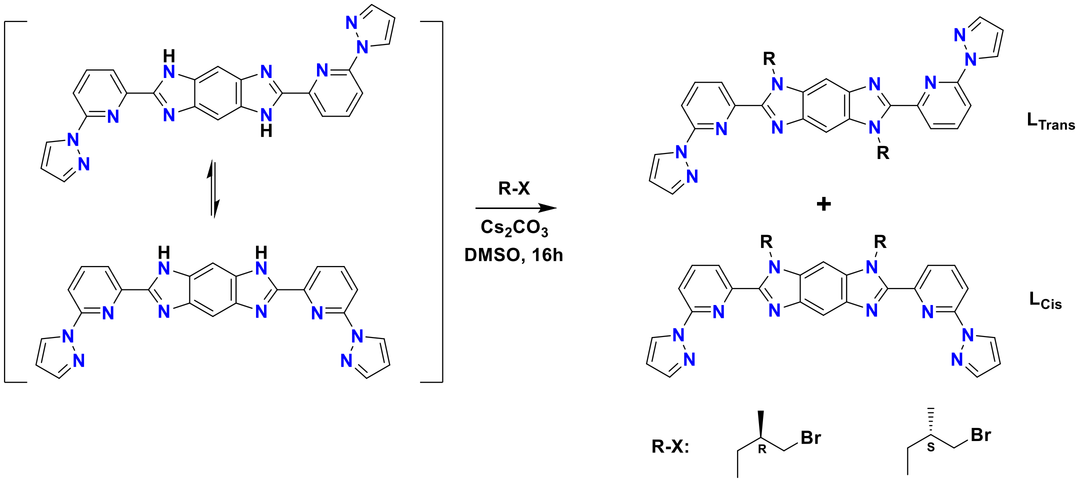

Figure SI-2: *Synthetic scheme for preparation of modified ligands* ***L$_S$*** *and* ***L$_R$****.*

2.1. 6-(pyrazol-1-yl)picolinic acid (A)

Pyrazole (6.2 g, 91.1 mmol, 2.1 eq) was dissolved in 1,4-dioxane (200 mL) in a 500 mL three-necked round-bottomed flask. The mixture was bubbled with argon for 30 min. Later potassium tertiary butoxide (10.7 g, 95.4 mmol, 2.2 eq) was added and stirred at room temperature under argon for 30 min. Later ethyl 6-bromopyridine-2-carboxylate (10.0 g, 43.5 mmol, 1 eq) was added and the mixture was stirred for 48 hours at 115 °C under Argon atmosphere. The reaction was stopped and cooled to room temperature. The solvent was evaporated under reduced pressure. The crude product was suspended and refluxed for 3 hours in EtOH (100 mL) and NaOH solution (2M, 50 mL). Ethanol was removed under reduced pressure and 50 mL water was added to the

mixture. The pH was adjusted to 3 with aq. HCl, which resulted in precipitation. The colorless precipitate was collected by filtration and dried at 80 °C in an oven.

Yield: 5.59 g (67.8 %)
ESI-MS, found (calcd): 188.0472 $[M-H]^-$, (188.0455); 144.0601 $[M-COOH]^-$, (144.0556).
IR (KBr, cm^{-1}): 3435, 3068, 2998, 2581, 1706, 1639, 1593, 1579, 1530, 1484, 1459, 1425, 1398, 1345,1331, 1315, 1267, 1211, 1162, 1136, 1074, 1055, 993, 948, 847, 836, 774, 761, 724, 651, 611, 589, 483.
^{1}H NMR (500 MHz, CD_3OD) δ/ppm: 13.03 (b, OH), 8.91 (d, 1H), 8.19 (d, 1H), 8.13 (t, 1H), 8.07 (d, 1H), 7.81 (d, 1H), 6.58 (dd, 1H).
^{13}C NMR (126 MHz, CD_3OD) δ/ppm: 166.48, 151.21, 147.04, 142.34, 140.23, 127.90, 122.50, 115.51, 107.93.
Elemental analysis for $C_9H_7N_3O_2$, found(calcd): C: 56.54 (57.14); H: 3.35 (3.73); N 22.01 (22.21).

2.2. 2,6-bis(6-(pyrazol-1-yl)pyridin-2-yl)-1,5-dihydrobenzo[1,2-d:4,5-d']diimidazole (L)

A (6-(pyrazol-1-yl)picolinic acid, 2.41 g, 12.75 mmol, 2.1 eq), 1,2,4,5-benzenetetramine tetra hydrochloride (1.72 g, 6.05 mmol, 1 eq) and polyphosphoric acid (20 mL) were taken in a round-bottomed flask. The reaction mixture was gently heated at 130 °C until the polyphosphoric acid got viscous enough to allow the stirring with a magnetic stirrer bar. Then the temperature was set to 200 °C and stirred for 4 hours. The reaction was cooled to 100 °C and was added into crushed ice. The aqueous suspensions were combined, and the precipitate was filtered off. The solid obtained was suspended in water, and the pH was adjusted to 10 using aq. Na_2CO_3 solution (2 M). After the suspension was stirred for 2 hours, it was collected by filtration. The precipitate was re-suspended in water, pH was set to 4 using HCl (1 M), and stirred overnight. The precipitate was filtered off by washing with water (250 mL) and the light brown color product obtained was dried in the oven at 120 °C.

Yield: 2.25 g (79 %)
ESI-MS, found (calcd): 445.1607 $[M+H]^+$, (445.1632); 467.1421 $[M+Na]^+$, (467.1452); 889.3089 $[2M+H]^+$, (889.3192).
IR (KBr, cm^{-1}): 3295, 3099, 1601, 1579, 1522, 1474, 1456, 1394, 1341, 1291, 1237, 1204, 1155, 1060, 1038, 993, 938, 885, 834, 807, 752. 702, 651, 591, 418.
^{1}H NMR (500 MHz, DMSO-d6) δ/ppm: 13.04 (s, 2H, NH-S), 12.97 (s, 2H, NH-C), 9.32 (d, 2H), 9.29 (d, 2H), 8.26 (d, 4H), 8.19 (dt, 4H), 8.07 (s, 1H, H_β), 8.02 (dd, 4H), 7.92 (s, 4H), 7.88 (s, 2H, H_α), 7.73 (s, 1H, H_γ), 6.74 (dd, 4H).
^{13}C NMR (126 MHz DMSO-d6) δ/ppm: 151.46, 150.99, 147.49, 143.12, 143.10, 142.20, 141.33, 134.04, 133.14, 128.72, 128.64, 119.22, 119.14, 112.59, 108.72, 108.63, 100.22, 92.40.

Elemental analysis for $C_{24}H_{18}N_{10}Cl_2$ (L·2HCl), found(calcd): C: 54.54 (55.72); H: 3.36 (3.51); N 27.91 (27.07).

2.3 Modified Ligand L_S

L (0.895 g, 2.0 mmol, 1 eq) and Cs_2CO_3 (2.64 g, 8 mmol, 4 eq) were taken in a three-necked round-bottom flask and dried under vacuum at 100 °C for three hours. The flask was flushed with argon, and DMSO (30 mL) was added. After 30 min, the suspension was allowed to cool to ambient temperature. The (S)-(+)-1-bromo-2-methylbutane (0.838 g, 6 mmol, 3 eq, V=0.566 mL) was added using a syringe and continued to stir at 80 °C under Argon. The reaction was stopped after 24 h. The reaction mixture was taken into a mixture of $CHCl_3$/EtOAc (1:1) and H_2O. After phase separation, the aqueous layer was extracted twice with $CHCl_3$. The organic layer was dried over Na_2SO_4, filtered, and evaporated under reduced pressure. Then the products were purified by silica gel column chromatography (1:24 MeOH/$CHCl_3$ v/v) using $CHCl_3$ as an initial eluent. (Length of the column = 35 cm, and diameter of the column used = 3 cm)

Trans (L_S)

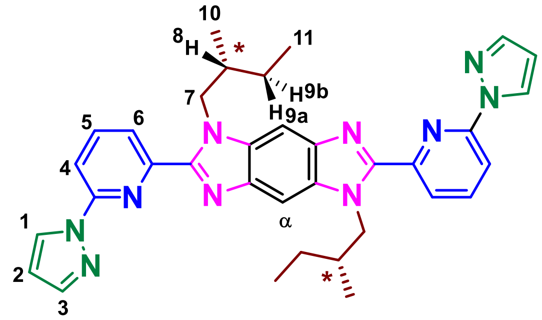

R_f = 0.63 (1:24 MeOH/$CHCl_3$ v/v)
Yield: 0.37 g (33 %)
$[\alpha]_D^{25}$= +7.39 (c = 0.00176 g/mL, DCM)
ESI-MS, found (calcd): 561.2464 $[M+H]^+$, (561.2469); 1121.4873 $[2M+H]^+$, (1121.4866).
IR (KBr, cm^{-1}): 3422, 3140, 3108, 2956, 2924, 2815, 1078, 1742, 1594, 1574, 1510, 1471, 1395, 1279, 1248, 1200, 1150, 1118, 1074, 1040, 969, 948, 906, 886, 812, 790, 754, 656, 626.
^{1}H NMR (500 MHz, CD_2Cl_2) δ/ppm: 8.63 (d, 2H, **1**), 8.39 (d, 2H, **4**), 8.11 (d, 2H, **6**), 8.05 (dd, 2H, **5**), 7.86 (s, 2H, Hα), 7.83 (d, 2H, **3**), 6.60 (dd, 2H, **2**), 4.95 (t, 4H, **7**), 2.20 (m, 2H, **8**), 1.46 (m, 2H, **9b**), 1.26(m, 2H, **9a**), 0.88(m, 12H, **10** & **11**).
^{13}C NMR (126 MHz, $CDCl_3$) δ/ppm: 150.86, 150.26, 148.40, 142.38, 140.63, 139.83, 135.46, 127.02, 122.50, 112.95, 108.36, 99.33, 71.13, 59.15, 45.44.
Elemental analysis for $C_{34}H_{26}N_{10}$, found(calcd): C: 69.42 (69.84); H: 6.10 (6.21); N 23.61 (23.95).

Cis – S

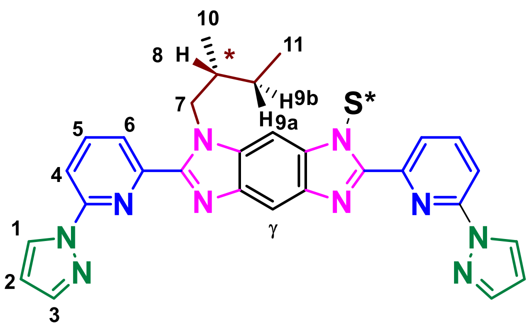

R_f = 0.47 (1:24 MeOH/$CHCl_3$ v/v)
Yield: 0.33 g (29 %)
ESI-MS, found (calcd): 561.2469 $[M+H]^+$, (561.2469)
IR (KBr, cm^{-1}): 3375, 3143, 3104, 2936, 2815, 2720, 1729, 1595, 1573, 1520, 1470, 1395, 1374, 1338, 1277, 1202, 1150, 1114, 1077, 1042, 994, 966, 946, 905,869, 812, 754, 660, 622.
^{1}H NMR (500 MHz, CD_2Cl_2) δ/ppm: 8.62 (d, 2H, **1**), 8.40 (d, 2H, **4**), 8.17 (s, 1H, **H$_\beta$**), 8.10 (dd, 2H, **6**), (8.05 t, 2H, **5**), 7.83 (d, 2H, **3**), 7.39 (s, 1H, **H$_\gamma$**), 6.60 (dd, 2H, **2**), 4.97 (t, 4H, **7**), 2.15 (m, 2H, **8**), 1.44(m, 2H, **9b**), 1.24(m, 2H, **9a**), 0.88(m, 12H, **10** and **11**).
^{13}C NMR (126 MHz, $CDCl_3$) δ/ppm: 150.79, 149.65, 148.61, 142.41, 140.32, 139.80, 136.15, 126.99, 122.43, 112.81, 109.48, 108.39, 89.91, 71.53, 59.27, 45.59.

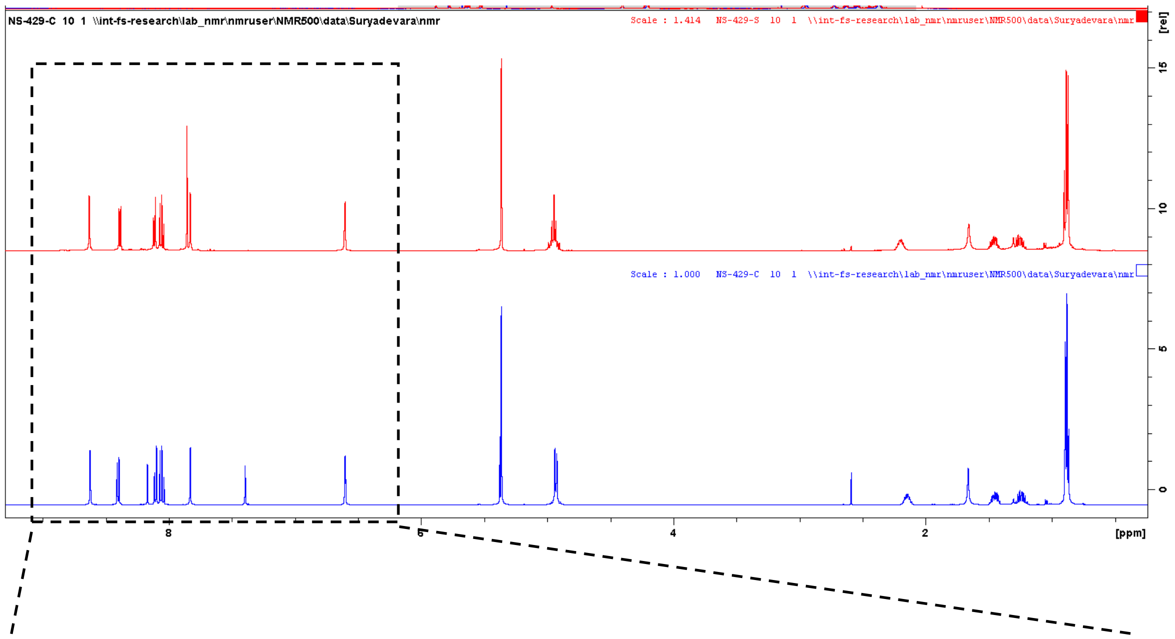

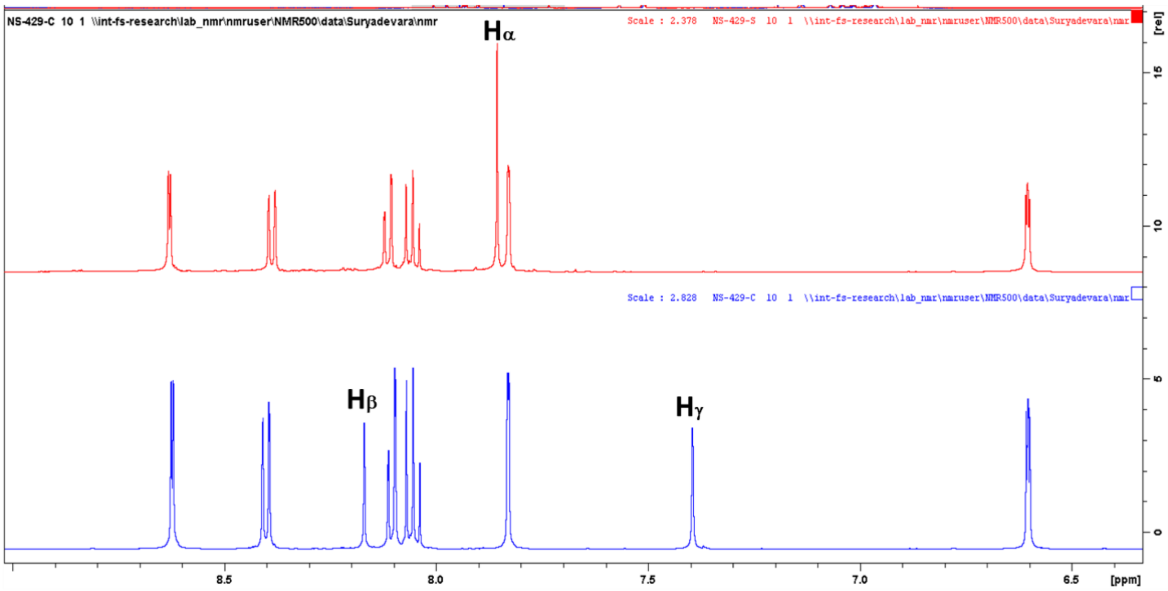

Figure SI-3: ^{1}H-NMR of ligand **L$_S$**.

2.4 Modified Ligand L$_R$

L (0.895 g, 2.0 mmol, 1 eq) and Cs_2CO_3 (2.64 g, 8 mmol, 4 eq) were taken in a three-necked round-bottomed flask and dried under vacuum at 100 °C for three hours. The flask was flushed with argon, and DMSO (30 mL) was added. After 30 min, the suspension was allowed to cool to ambient temperature. The (R)-(-)-1-bromo-2-methylbutane (0.838 g, 6 mmol, 3 eq, V=0.566 mL) was added using a syringe and continued to stir at 80 °C under argon. The reaction was stopped after 24 h. The reaction mixture was taken into a mixture of $CHCl_3$/EtOAc (1:1) and H_2O. After phase separation, the aqueous layer was extracted twice with $CHCl_3$. The organic layer was dried over Na_2SO_4, filtered, and evaporated under reduced pressure. Then the products were purified by silica gel column chromatography (1:24 MeOH/$CHCl_3$ v/v) using $CHCl_3$ as an initial eluent. (Length of the column = 35 cm, and diameter of the column used = 3 cm)

Trans (L$_R$)

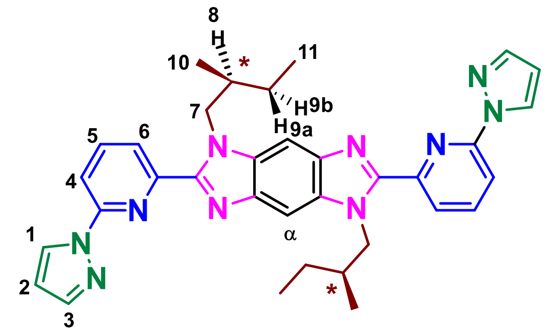

R_f = 0.63 (1:24 MeOH/CHCl$_3$ v/v)
Yield: 0.37 g (33 %)
$[\alpha]_D^{25}$= -6.63 (c = 0.0016 g/mL, DCM)
ESI-MS, found (calcd): 561.2464 $[M+H]^+$, (561.2469); 1121.4873 $[2M+H]^+$, (1121.4866).
IR (KBr, cm^{-1}): 3422, 3140, 3108, 2956, 2924, 2815, 1078, 1742, 1594, 1574, 1510, 1471, 1395, 1279, 1248, 1200, 1150, 1118, 1074, 1040, 969, 948, 906, 886, 812, 790, 754, 656, 626.
^{1}H NMR (500 MHz, CD$_2$Cl$_2$) δ/ppm: 8.62 (d, 2H, **1**), 8.39 (d, 2H, **4**), 8.12 (d, 2H, **6**), 8.06 (dd, 2H, **5**), 7.86 (s, 2H, Hα), 7.83 (d, 2H, **3**), 6.60 (dd, 2H, **2**), 4.95 (t, 4H, **7**), 2.20 (m, 2H, **8**), 1.46 (m, 2H, **9a**), 1.26(m, 2H, **9b**), 0.89(m, 12H, **10** & **11**).
^{13}C NMR (126 MHz, CDCl$_3$) δ/ppm: 150.86, 150.26, 148.40, 142.38, 140.63, 139.83, 135.46, 127.02, 122.50, 112.95, 108.36, 99.33, 71.13, 59.15, 45.44.
Elemental analysis for $C_{34}H_{26}N_{10}$, found(calcd): C: 69.47 (69.84); H: 6.33 (6.21); N 23.21 (23.95).

Cis – R

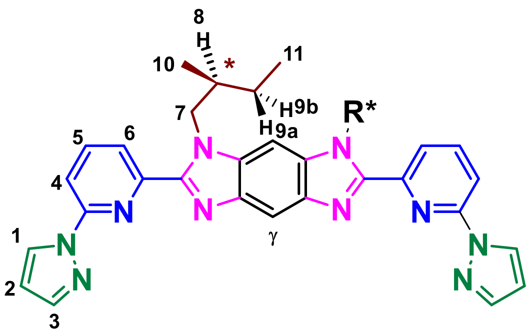

R_f = 0.47 (1:24 MeOH/CHCl$_3$ v/v)
Yield: 0.33 g (29 %)
ESI-MS, found (calcd): 561.2469 $[M+H]^+$, (561.2469)
IR (KBr, cm^{-1}): 3375, 3143, 3104, 2936, 2815, 2720, 1729, 1595, 1573, 1520, 1470, 1395, 1374, 1338, 1277, 1202, 1150, 1114, 1077, 1042, 994, 966, 946, 905,869, 812, 754, 660, 622.
^{1}H NMR (500 MHz, CD$_2$Cl$_2$) δ/ppm: 8.62 (d, 2H, **1**), 8.40 (d, 2H, **4**), 8.17 (s, 1H, **H$_β$**), 8.10 (dd, 2H, **6**), 8.05 (t, 2H, **5**), 7.83 (d, 2H, **3**), 7.39 (s, 1H, H$_γ$), 6.60 (dd, 2H, **2**), 4.97 (t, 4H, **7**), 2.15 (m, 2H, **8**), 1.44(m, 2H, **9a**), 1.24(m, 2H, **9b**), 0.88(m, 12H, **10** & **11**).^{13}C NMR (126 MHz, CDCl$_3$) δ/ppm: 150.79, 149.65, 148.61, 142.41, 140.32, 139.80, 136.15, 126.99, 122.43, 112.81, 109.48, 108.39, 89.91, 71.53, 59.27, 45.59.

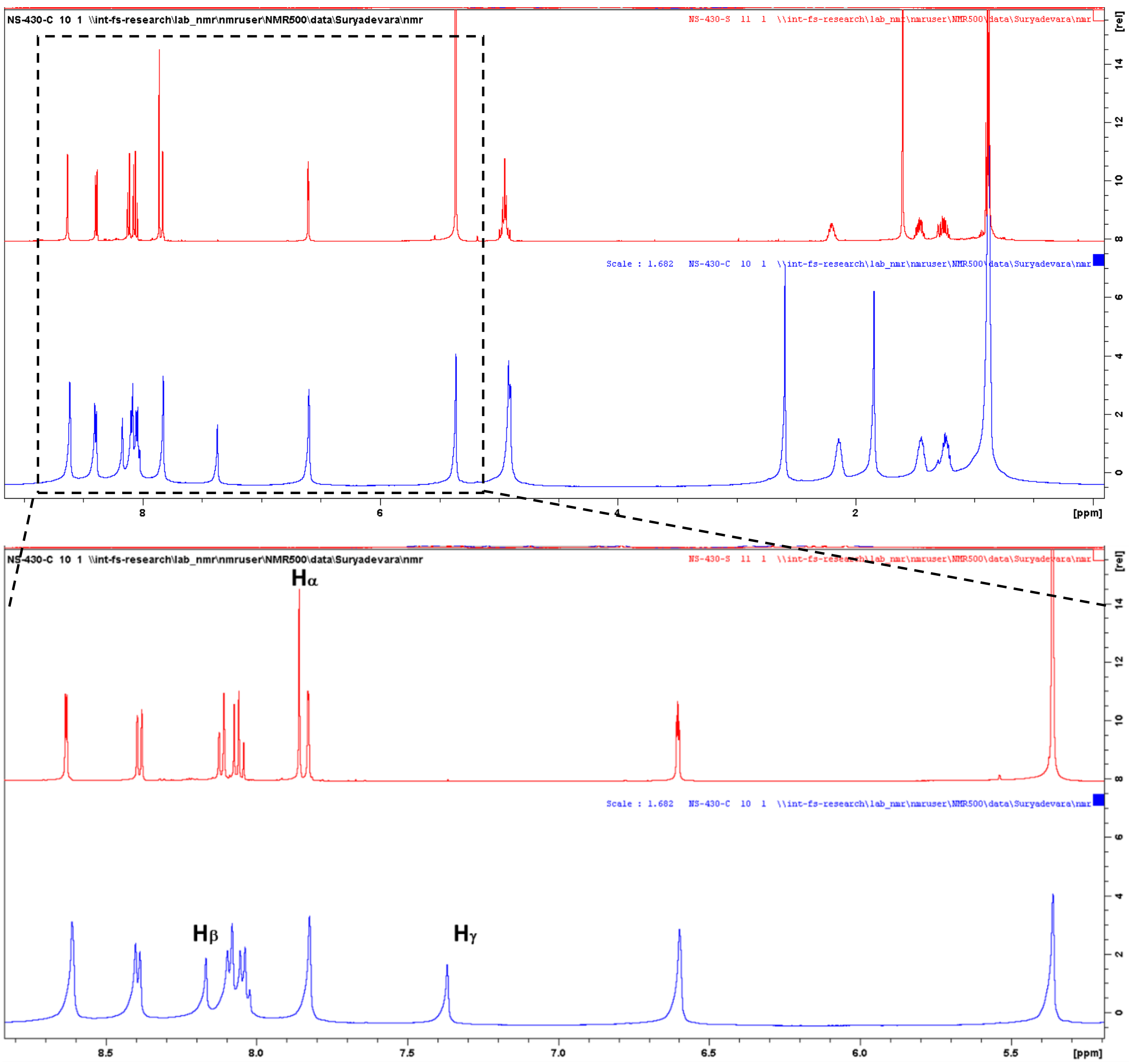

Figure SI-4: ^{1}H-NMR of ligand $\mathbf{L_R}$.

2. Synthesis of [2x2]-iron(II) grid complexes

Synthesis of P_S

$\mathbf{L_S}$ (0.20 g, 0.35 mmol, 1 eq.) was suspended in dry acetonitrile (60 mL) sonicated for 30 min and then degassed with argon for 30 min. Afterwards $Fe(CF_3SO_3)_2$ (anhydrous) (0.12 g, 0.35 mmol, 1 eq.) was added. The reaction was stirred at 60 °C for 14 hours under Ar atmosphere. The reaction mixture was cooled to room temperature and filtered. The filtrate was reduced to about 5 mL. The product was precipitated by the addition of diethyl ether (30 mL) and collected by centrifugation (4000 Hz, 10 min). The ether was removed using a pipette. The dark maroon coloured precipitate was dried under vacuum.

Yield: 0.22 g (69 %)

Synthesis of P_R

L_R (0.20 g, 0.35 mmol, 1 eq.) was suspended in dry acetonitrile (60 mL), sonicated for 30 min and then degassed with argon for 30 min. Afterwards $Fe(CF_3SO_3)_2$ (anhydrous) (0.12 g, 0.35 mmol, 1 eq.) was added. The reaction was stirred for 14 hours at 60 °C under Ar atmosphere. It was cooled to room temperature and filtered. The filtrate was reduced to about 5 mL. The product was precipitated by the addition of diethyl-ether (30 mL) and collected by centrifugation (4000 Hz, 10 min). The ether was removed using a pipette. The dark maroon colored precipitate was dried at vacuum.

Yield: 0.285 g (89 %)

Synthesis of $^{57}FeTf_2$ (anhydrous):

^{57}Fe powder (200 mg, 3.51 mmol) was taken in dry acetonitrile (3 mL) in a 10 mL round bottomed flask. Triflic acid (0.7 mL) was added slowly dropwise, as the reaction is highly exothermic. After the initial effervescence subsided, the mixture was heated to 60 °C and maintained under Ar. Once the effervescence had stopped, the mixture was cooled to RT and unreacted Fe powder was filtered through celite pad. The filtrate was concentrated under reduced pressure and stored at -20 °C over night which resulted in colourless crystals. The crystals were filtered and by washing with DEE and dried under vacuum overnight to remove solvent.

Yield: 550 mg (43%)

Elemental Analysis for $^{57}FeC_2F_6O_6S_2$ found (calcd): C: 6.39 (6.79); S: 18.44 (18.12).

3. ESI-MS and IMMS:

Experimental Instrument Parameters: About 10 μg/mL concentrated solution in acetonitrile was directly electrosprayed. A Waters Synapt G2S HDMS high-resolution mass spectrometer coupled with ion mobility separation cell was used for this purpose. The optimized parameters are as follows:

Polarity: ES+, Capillary voltage: 1.5 kV, Source Temperature: 50°C, Sampling Cone: 20 V, Source Offset: 30 V, Source Gas Flow: 0 mL/min, Desolvation Temperature: 120°C, Cone Gas Flow: 0 L/h, Desolvation Gas Flow: 650.0 L/h, Nebuliser Gas Flow: 2.5 bar, Trap Gas Flow: 10.00 mL/min, Helium Gas Flow: 200 mL/min, IMS Gas Flow: 60 mL/min, IMS Wave Velocity: 500 m/s, IMS Wave Height: 35 V

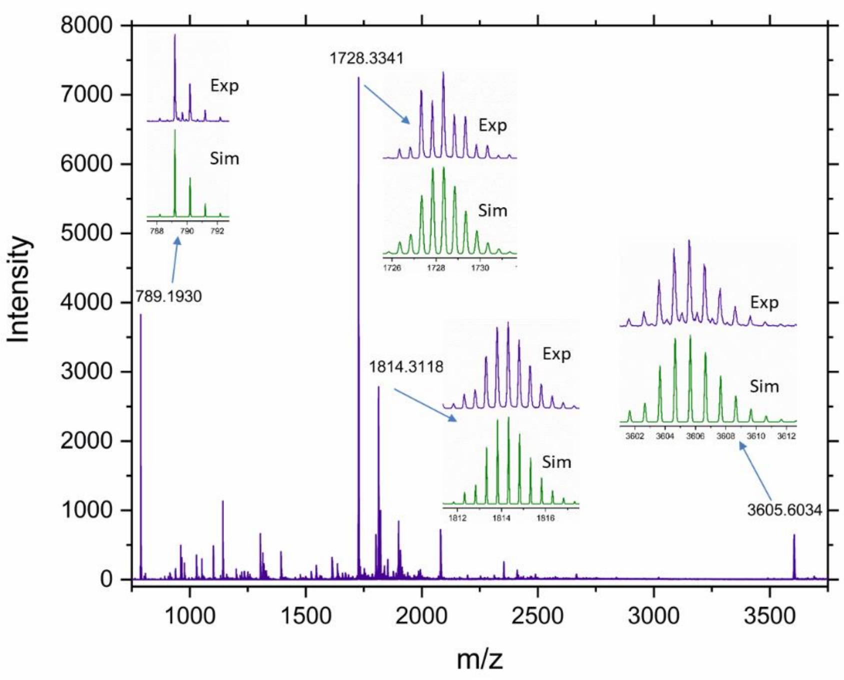

Figure SI-5: *ESI data of the grid complex in acetontrile.*

m/z	Charge	Formula
3605.6034	1	$Fe_4L_4(Tf)_7$
1814.3118	2	$Fe_4L_4(Tf)_7Na$
1728.3341	2	$Fe_4L_4(Tf)_6$
789.1920	1	FeL(Tf)

Table SI-1: *ESI peaks (L: $C_{34}H_{34}N_{10}$, Tf:CF_3SO_3)*

4. Circular dichroism (CD) of grid complexes

A Jasco J-815 CD Spectrometer (Jasco, Inc.) was used with Spectra Manager software (Jasco Inc.) to record CD spectra. Solutions were prepared by dissolving the samples in acetonitrile to a concentration of 10^{-6} M. All spectra were recorded at 25 °C between 700 and 200 nm at a rate of 50 nm/min and were averaged to 2 accumulations. A 1.0 mm path length cuvette was used to hold solutions, and all spectra were background-subtracted from those of the mobile phases.

5. Deconvolution of diastereomeric grid complexes

Diastereomers in each sample were separated by performing recrystallization several times. In first step of crystallization, 150 mg of sample was dissolved in *ca.* 15 mL of acetonitrile, and THF was added slowly via vapor diffusion. This yielded in the crystalline sample. The crystals were dried, powdered and again dissolved in acetonitrile. THF was slowly diffused again to yield the second recrystallized sample. In each step, the finely powdered sample was used for measuring CD. The powder sample was used instead of crystals to maintain the uniformity. After four steps, the saturation of the CD signal in each case was observed.

Yield for Λ_S-isomer after four recrystallizations: 40% (with respect to the weight of crude sample used for recrystallization)
Yield on 1st step of recrystallization: 70.8%
Yield on 2nd step of recrystallization: 86.5%
Yield on 3rd step of recrystallization: 81.6%
Yield on Final (4th) step of recrystallization: 80.0%
IR (KBr, cm^{-1}): 3494, 3107, 2966, 2879, 1608, 1568, 1517, 1493, 1472, 1447, 1422, 1408, 1374, 1357, 1252, 1221, 1197, 1142, 1093, 1050, 1027, 965, 933, 901, 796, 754, 634, 572, 516.
Elemental Analysis for $Fe_4C_{144}H_{144}N_{40}F_{24}O_{24}S_8$, found (calcd): C: 45.73 (46.06); H: 3.67 (3.87); N: 14.50 (14.92); S: 6.14 (6.83).

Yield for Δ_R -isomer after four recrystallizations: 45% (with respect to the weight of crude sample used for recrystallization)
Yield on 1st step of recrystallization: 76.6%
Yield on 2nd step of recrystallization: 77.0%
Yield on 3rd step of recrystallization: 82.0%
Yield on Final (4th) step of recrystallization: 81.7%
IR (KBr, cm^{-1}): 3495, 3107, 2966, 2879, 1608, 1568, 1517, 1493, 1472, 1447, 1422, 1408, 1374, 1357, 1251, 1221, 1197, 1141, 1093, 1050, 1026, 965, 934, 901, 796, 754, 634, 572, 515, 460.
Elemental Analysis for $Fe_4C_{144}H_{144}N_{40}F_{24}O_{24}S_8$, found (calcd): C: 45.69 (46.06); H: 3.90 (3.87); N: 14.43 (14.92); S: 6.04 (6.83).

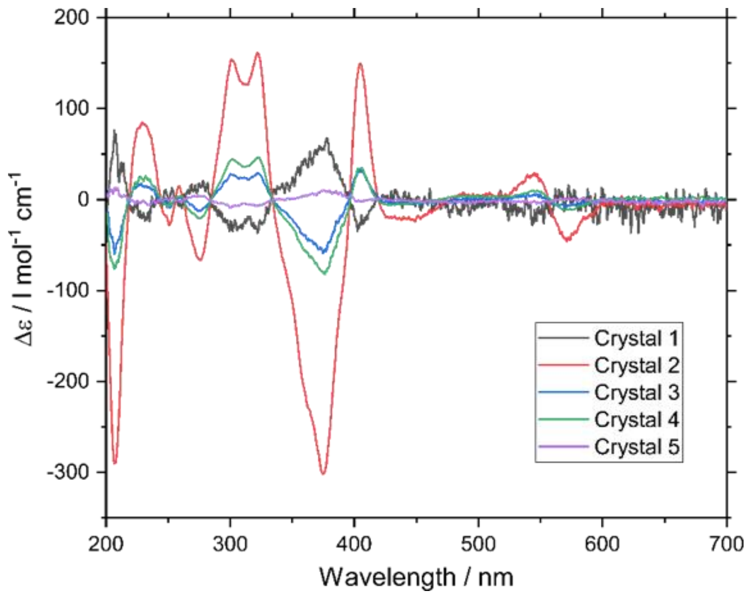

Figure SI-6: *CD spectra of different single crystals grown in a crystallization vial by vapor diffusion of THF into complex in acetonitrile. These crystals were formed during first crystallization from Iron(II) complex prepared from ligand L_S.*

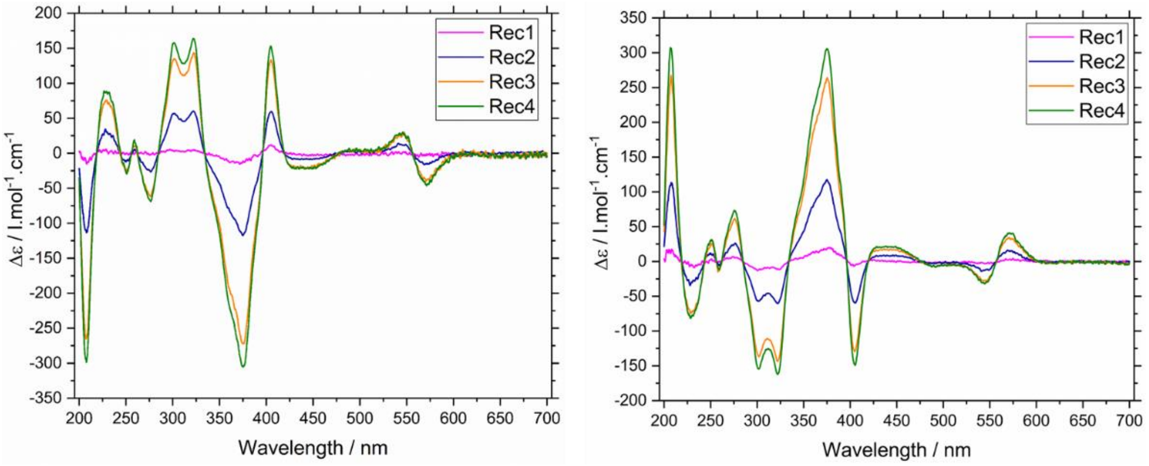

Figure SI-7: *CD spectra over 4 recrystallization steps, each recrystallization step by vapour diffusion of THF into complex dissolved in acetonitrile for complex formed - (Λ_S) isomer using ligand L_S (on left) and (Δ_R) isomer using ligand L_R(on right)*

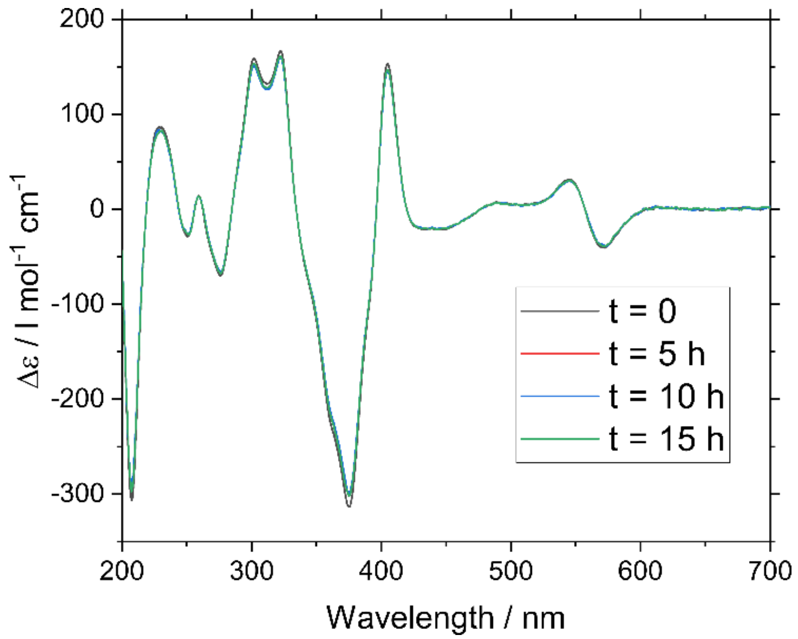

Figure SI-8: *Time dependent spectra of Λ_S-isomer measured over a period of 15 hours.*

6. X-ray diffraction analysis

The single-crystal X-ray diffraction data of the complexes Λ_S and Δ_R were collected using STOE Stadi Vari 25 diffractometer with graded multilayer mirrored monochromatic Ga-Kα radiation (λ = 1.34143 Å).

The structures were solved using direct methods and were refined by full-matrix least square methods and using SHELX-2014 inbuilt in Olex2. Data were collected using ϕ and Ω scans chosen to complete the asymmetric unit. All non-hydrogen atoms were refined anisotropically, whereas the hydrogen atoms were calculated geometrically, riding on their parent atoms. Since the crystals were weakly diffracting, and thus due to poor quality of the data, only four of eight anions could be located. Also, all well diffracting crystals were found to be the pure diastereomers. We expect the other crystals could contain various ratio of diastereomers, which could account for the poor diffraction profile. Our attempts to determine the X-ray structure of the mixed crystals were not successful due to the inferior quality of the crystals.

Crystallographic data for the structures reported in this paper have been deposited with the Cambridge Crystallographic Data Centre as supplementary publication no. CCDC-2064448 and 2064449. Copies of the data can be obtained free of charge from www.ccdc.cam.ac.uk/conts/retrieving.html.

Table SI-2: Complex Λ_S and Δ_R:

	Λ_S	Δ_R
Formula	$C_{140}H_{144}F_{12}Fe_4N_{40}O_{12}S_4$	$C_{140}H_{144}F_{12}Fe_4N_{40}O_{12}S_4$
Formula weight / g mol^{-1}	3158.59	3158.59
Temperature / K	180	180
Crystal dimensions / mm^3	0.15 × 0.06 × 0.04	0.1 × 0.07 × 0.07
System	Tetragonal	Tetragonal
Space group	*I*4 (#79)	*I*4 (#79)
a / Å	20.0596(6)	20.1380(5)
b / Å	20.0596(6)	20.1380(5)
c / Å	24.4391(9)	24.8755(7)
V / Å^3	9834.0(7)	10088.0(6)
Z	2	2
ρ_{calcd} / g cm^{-3}	1.067	1.040
μ (GaK$_\alpha$) / mm^{-1}	2.181	2.126
λ / Å	1.34143	1.34143
$2\theta_{max}$ / °	118.27	116.98
Reflections collected	18746	26886
Unique reflections (R_{int})	9873 (0.0430)	10325 (0.0356)
Number of parameters	523	536
Number of restraints	58	90
Final R_1 [$I > 2\sigma(I)$]	0.0685	0.0770
wR_2 (all data)	0.2151	0.2562
Goodness of fit	0.995	1.090
Largest diff. peak/ hole / e$^-$ Å^{-3}	0.50/-0.36	0.62/-0.60
Flack parameter	0.056(17)	0.12(2)
Hooft parameter	0.010(6)	0.037(13)
Void volume	36.7%	36.7%
CCDC Deposition Number	2064448	2064449

Table SI-3: Bond lengths and angular parameters of the two enantiomeric grid complexes Λ_S and Δ_R:

**Parameters**	Λ_S	Δ_R
Temperature / K	180	180
Avg. Fe-N Bond length /Å	1.941(2)	1.951(2)
N_{py}-Fe-N_{py} (ϕ)/ °	175.25(3)	175.14(7)
Σ / °	98.46(8)	98.13(9)
a=b / Å	20.0596(6)	20.0874(7)
c / Å	24.4391(9)	24.6612(6)

7. TDDFT Calculations

TDDFT calculations on the Λ_S-complex were performed in order to study its CD spectrum using the escf module[1,2,3] of the Turbomole package (Version 7.4.1)[4]. For this, we used the PBE0 functional[5,6] together with the def2-SV(P)[7] basis set for all atoms except for iron for which the def2-TZVP[7] basis set was chosen. The exchange-correlation functional was evaluated using a gridsize of 5, as implemented in Turbomole.[8]

Coordinates of optimized structure of Λ_S (HS complex)

324
Energy =

Fe	6.6894339	1.0194184	-0.0421541
N	8.3892918	-0.2500012	-0.5855511
N	8.7604181	-0.1436057	-1.8801616
N	6.8979415	1.1676327	-2.1948433
N	5.0433241	2.3885045	-0.7620577
N	3.9537943	3.3296363	-2.4789187
N	0.7618126	5.5119099	0.6852381
N	1.9909764	4.7619523	2.4018871
N	-0.9482092	6.9305673	2.1117443
N	-2.7406098	8.3342108	1.7905959
N	-2.7231661	7.9563466	0.4938271
C	9.3258011	-0.9822928	-0.0001921
H	9.2590635	-1.2139843	1.0633017
C	10.3267327	-1.3621660	-0.9181480
H	11.2216153	-1.9557298	-0.7318563
C	9.9324331	-0.8065022	-2.1115575
H	10.4067236	-0.8344513	-3.0918398
C	7.9703906	0.6141004	-2.7512985
C	8.2957674	0.7933238	-4.1015844
H	9.1833824	0.3433489	-4.5500840
C	7.4488466	1.5917360	-4.8539548
H	7.6705205	1.7717887	-5.9101164
C	6.3242982	2.1852758	-4.2673659
H	5.6931022	2.8238625	-4.8802703
C	6.0754426	1.9581962	-2.9139824
C	5.0044539	2.5505862	-2.0817034
C	3.2716146	3.6993402	-1.3307864
C	2.1351254	4.4874035	-1.1559716
H	1.5674924	4.9157309	-1.9834134
C	1.7772967	4.7373438	0.1718302
C	2.5507158	4.2246558	1.2535846
C	3.6486313	3.3838357	1.0789065
H	4.2287185	2.9734999	1.9067180
C	3.9961169	3.1195321	-0.2488953
C	0.9278202	5.5242051	2.0049052

C	0.0371316	6.3634935	2.8373121
C	0.1594710	6.6458516	4.1977861
H	0.9396495	6.2131228	4.8188344
C	-0.7418211	7.5439511	4.7827620
H	-0.6503679	7.7926152	5.8442792
C	-1.7381333	8.1359543	4.0229765
H	-2.4316473	8.8502858	4.4704906
C	-1.7967879	7.7890313	2.6674050
C	-3.6888370	8.6455614	-0.0964988
H	-3.8818730	8.5211800	-1.1625347
C	-4.3497298	9.4888274	0.8204880
H	-5.1742563	10.1758109	0.6306073
C	-3.7154583	9.2638653	2.0186311
H	-3.8880907	9.7039841	3.0000500
C	2.5590913	4.5842175	3.7361703
H	2.6579537	5.5748583	4.2053843
H	3.5931541	4.2358474	3.5892958
C	1.8104324	3.6029785	4.6445919
H	0.7493794	3.9193497	4.6922120
C	2.3948947	3.7046510	6.0605939
H	2.3347221	4.7577015	6.3953600
H	3.4768280	3.4709966	6.0209465
C	1.7212110	2.8210121	7.1012740
H	0.6299717	2.9929471	7.1352844
H	2.1170483	3.0393364	8.1065882
H	1.8917131	1.7469514	6.9124450
C	1.8458396	2.1858366	4.0806178
H	1.4671365	2.1452126	3.0435195
H	1.2234604	1.5058662	4.6849425
H	2.8772673	1.7848497	4.0867892
Fe	-1.0194184	6.6894339	-0.0421541
N	0.2500012	8.3892918	-0.5855511
N	0.1436057	8.7604181	-1.8801616
N	-1.1676327	6.8979415	-2.1948433
N	-2.3885045	5.0433241	-0.7620577
N	-3.3296363	3.9537943	-2.4789187
N	-5.5119099	0.7618126	0.6852381
N	-4.7619523	1.9909764	2.4018871
N	-6.9305673	-0.9482092	2.1117443
N	-8.3342108	-2.7406098	1.7905959
N	-7.9563466	-2.7231661	0.4938271
C	0.9822928	9.3258011	-0.0001921
H	1.2139843	9.2590635	1.0633017
C	1.3621660	10.3267327	-0.9181480
H	1.9557298	11.2216153	-0.7318563
C	0.8065022	9.9324331	-2.1115575
H	0.8344513	10.4067236	-3.0918398
C	-0.6141004	7.9703906	-2.7512985
C	-0.7933238	8.2957674	-4.1015844
H	-0.3433489	9.1833824	-4.5500840
C	-1.5917360	7.4488466	-4.8539548

H	-1.7717887	7.6705205	-5.9101164
C	-2.1852758	6.3242982	-4.2673659
H	-2.8238625	5.6931022	-4.8802703
C	-1.9581962	6.0754426	-2.9139824
C	-2.5505862	5.0044539	-2.0817034
C	-3.6993402	3.2716146	-1.3307864
C	-4.4874035	2.1351254	-1.1559716
H	-4.9157309	1.5674924	-1.9834134
C	-4.7373438	1.7772967	0.1718302
C	-4.2246558	2.5507158	1.2535846
C	-3.3838357	3.6486313	1.0789065
H	-2.9734999	4.2287185	1.9067180
C	-3.1195321	3.9961169	-0.2488953
C	-5.5242051	0.9278202	2.0049052
C	-6.3634935	0.0371316	2.8373121
C	-6.6458516	0.1594710	4.1977861
H	-6.2131228	0.9396495	4.8188344
C	-7.5439511	-0.7418211	4.7827620
H	-7.7926152	-0.6503679	5.8442792
C	-8.1359543	-1.7381333	4.0229765
H	-8.8502858	-2.4316473	4.4704906
C	-7.7890313	-1.7967879	2.6674050
C	-8.6455614	-3.6888370	-0.0964988
H	-8.5211800	-3.8818730	-1.1625347
C	-9.4888274	-4.3497298	0.8204880
H	-10.1758109	-5.1742563	0.6306073
C	-9.2638653	-3.7154583	2.0186311
H	-9.7039841	-3.8880907	3.0000500
C	-4.5842175	2.5590913	3.7361703
H	-5.5748583	2.6579537	4.2053843
H	-4.2358474	3.5931541	3.5892958
C	-3.6029785	1.8104324	4.6445919
H	-3.9193497	0.7493794	4.6922120
C	-3.7046510	2.3948947	6.0605939
H	-4.7577015	2.3347221	6.3953600
H	-3.4709966	3.4768280	6.0209465
C	-2.8210121	1.7212110	7.1012740
H	-2.9929471	0.6299717	7.1352844
H	-3.0393364	2.1170483	8.1065882
H	-1.7469514	1.8917131	6.9124450
C	-2.1858366	1.8458396	4.0806178
H	-2.1452126	1.4671365	3.0435195
H	-1.5058662	1.2234604	4.6849425
H	-1.7848497	2.8772673	4.0867892
Fe	1.0194184	-6.6894339	-0.0421541
N	-0.2500012	-8.3892918	-0.5855511
N	-0.1436057	-8.7604181	-1.8801616
N	1.1676327	-6.8979415	-2.1948433
N	2.3885045	-5.0433241	-0.7620577
N	3.3296363	-3.9537943	-2.4789187
N	5.5119099	-0.7618126	0.6852381

N	4.7619523	-1.9909764	2.4018871
N	6.9305673	0.9482092	2.1117443
N	8.3342108	2.7406098	1.7905959
N	7.9563466	2.7231661	0.4938271
C	-0.9822928	-9.3258011	-0.0001921
H	-1.2139843	-9.2590635	1.0633017
C	-1.3621660	-10.3267327	-0.9181480
C	-0.8065022	-9.9324331	-2.1115575
H	-0.8344513	-10.4067236	-3.0918398
C	0.6141004	-7.9703906	-2.7512985
C	0.7933238	-8.2957674	-4.1015844
H	0.3433489	-9.1833824	-4.5500840
C	1.5917360	-7.4488466	-4.8539548
H	1.7717887	-7.6705205	-5.9101164
C	2.1852758	-6.3242982	-4.2673659
H	2.8238625	-5.6931022	-4.8802703
C	1.9581962	-6.0754426	-2.9139824
C	2.5505862	-5.0044539	-2.0817034
C	3.6993402	-3.2716146	-1.3307864
C	4.4874035	-2.1351254	-1.1559716
H	4.9157309	-1.5674924	-1.9834134
C	4.7373438	-1.7772967	0.1718302
C	4.2246558	-2.5507158	1.2535846
C	3.3838357	-3.6486313	1.0789065
H	2.9734999	-4.2287185	1.9067180
C	3.1195321	-3.9961169	-0.2488953
C	5.5242051	-0.9278202	2.0049052
C	6.3634935	-0.0371316	2.8373121
C	6.6458516	-0.1594710	4.1977861
H	6.2131228	-0.9396495	4.8188344
C	7.5439511	0.7418211	4.7827620
H	7.7926152	0.6503679	5.8442792
C	8.1359543	1.7381333	4.0229765
H	8.8502858	2.4316473	4.4704906
C	7.7890313	1.7967879	2.6674050
C	8.6455614	3.6888370	-0.0964988
H	8.5211800	3.8818730	-1.1625347
C	9.4888274	4.3497298	0.8204880
H	10.1758109	5.1742563	0.6306073
C	9.2638653	3.7154583	2.0186311
H	9.7039841	3.8880907	3.0000500
C	4.5842175	-2.5590913	3.7361703
H	5.5748583	-2.6579537	4.2053843
H	4.2358474	-3.5931541	3.5892958
C	3.6029785	-1.8104324	4.6445919
H	3.9193497	-0.7493794	4.6922120
C	3.7046510	-2.3948947	6.0605939
H	4.7577015	-2.3347221	6.3953600
H	3.4709966	-3.4768280	6.0209465
C	2.8210121	-1.7212110	7.1012740
H	2.9929471	-0.6299717	7.1352844

H	3.0393364	-2.1170483	8.1065882
H	1.7469514	-1.8917131	6.9124450
C	2.1858366	-1.8458396	4.0806178
H	2.1452126	-1.4671365	3.0435195
H	1.5058662	-1.2234604	4.6849425
H	1.7848497	-2.8772673	4.0867892
Fe	-6.6894339	-1.0194184	-0.0421541
N	-8.3892918	0.2500012	-0.5855511
N	-8.7604181	0.1436057	-1.8801616
N	-6.8979415	-1.1676327	-2.1948433
N	-5.0433241	-2.3885045	-0.7620577
N	-3.9537943	-3.3296363	-2.4789187
N	-0.7618126	-5.5119099	0.6852381
N	-1.9909764	-4.7619523	2.4018871
N	0.9482092	-6.9305673	2.1117443
N	2.7406098	-8.3342108	1.7905959
N	2.7231661	-7.9563466	0.4938271
C	-9.3258011	0.9822928	-0.0001921
H	-9.2590635	1.2139843	1.0633017
C	-10.3267327	1.3621660	-0.9181480
H	-11.2216153	1.9557298	-0.7318563
C	-9.9324331	0.8065022	-2.1115575
H	-10.4067236	0.8344513	-3.0918398
C	-7.9703906	-0.6141004	-2.7512985
C	-8.2957674	-0.7933238	-4.1015844
H	-9.1833824	-0.3433489	-4.5500840
C	-7.4488466	-1.5917360	-4.8539548
H	-7.6705205	-1.7717887	-5.9101164
C	-6.3242982	-2.1852758	-4.2673659
H	-5.6931022	-2.8238625	-4.8802703
C	-6.0754426	-1.9581962	-2.9139824
C	-5.0044539	-2.5505862	-2.0817034
C	-3.2716146	-3.6993402	-1.3307864
C	-2.1351254	-4.4874035	-1.1559716
H	-1.5674924	-4.9157309	-1.9834134
C	-1.7772967	-4.7373438	0.1718302
C	-2.5507158	-4.2246558	1.2535846
C	-3.6486313	-3.3838357	1.0789065
H	-4.2287185	-2.9734999	1.9067180
C	-3.9961169	-3.1195321	-0.2488953
C	-0.9278202	-5.5242051	2.0049052
C	-0.0371316	-6.3634935	2.8373121
C	-0.1594710	-6.6458516	4.1977861
H	-0.9396495	-6.2131228	4.8188344
C	0.7418211	-7.5439511	4.7827620
H	0.6503679	-7.7926152	5.8442792
C	1.7381333	-8.1359543	4.0229765
H	2.4316473	-8.8502858	4.4704906
C	1.7967879	-7.7890313	2.6674050
C	3.6888370	-8.6455614	-0.0964988
H	3.8818730	-8.5211800	-1.1625347

C	4.3497298	-9.4888274	0.8204880
H	5.1742563	-10.1758109	0.6306073
C	3.7154583	-9.2638653	2.0186311
H	3.8880907	-9.7039841	3.0000500
C	-2.5590913	-4.5842175	3.7361703
H	-2.6579537	-5.5748583	4.2053843
H	-3.5931541	-4.2358474	3.5892958
C	-1.8104324	-3.6029785	4.6445919
H	-0.7493794	-3.9193497	4.6922120
C	-2.3948947	-3.7046510	6.0605939
H	-2.3347221	-4.7577015	6.3953600
H	-3.4768280	-3.4709966	6.0209465
C	-1.7212110	-2.8210121	7.1012740
H	-0.6299717	-2.9929471	7.1352844
H	-2.1170483	-3.0393364	8.1065882
H	-1.8917131	-1.7469514	6.9124450
C	-1.8458396	-2.1858366	4.0806178
H	-1.4671365	-2.1452126	3.0435195
H	-1.2234604	-1.5058662	4.6849425
H	-2.8772673	-1.7848497	4.0867892
H	-1.9557298	-11.2216153	-0.7318563
C	-3.5919669	-3.7934643	-3.8165054
C	-2.9202006	-2.7387761	-4.7028370
C	-2.8120734	-3.2422805	-6.1525016
C	-1.9543063	-4.4821425	-6.3802215
C	-1.5824648	-2.2866031	-4.1236383
H	-4.4895888	-4.2142674	-4.2955949
H	-2.9182435	-4.6504309	-3.6710960
H	-3.5830768	-1.8520426	-4.7253318
H	-0.8641049	-3.1230114	-4.0535286
H	-1.6963746	-1.8536556	-3.1136436
H	-1.1288854	-1.5135158	-4.7672560
H	-2.3444581	-5.3707211	-5.8522020
H	-3.8300419	-3.4368750	-6.5402425
H	-2.4207112	-2.4125606	-6.7680433
H	-1.9384643	-4.7367554	-7.4528241
H	-0.9056075	-4.3269074	-6.0689698
C	3.7934643	-3.5919669	-3.8165054
C	3.5919669	3.7934643	-3.8165054
C	-3.7934643	3.5919669	-3.8165054
C	2.7387761	-2.9202006	-4.7028370
C	2.9202006	2.7387761	-4.7028370
C	-2.7387761	2.9202006	-4.7028370
C	3.2422805	-2.8120734	-6.1525016
C	2.8120734	3.2422805	-6.1525016
C	-3.2422805	2.8120734	-6.1525016
C	4.4821425	-1.9543063	-6.3802215
C	1.9543063	4.4821425	-6.3802215
C	-4.4821425	1.9543063	-6.3802215
C	2.2866031	-1.5824648	-4.1236383
C	1.5824648	2.2866031	-4.1236383

C	-2.2866031	1.5824648	-4.1236383
H	4.2142674	-4.4895888	-4.2955949
H	4.4895888	4.2142674	-4.2955949
H	-4.2142674	4.4895888	-4.2955949
H	4.6504309	-2.9182435	-3.6710960
H	2.9182435	4.6504309	-3.6710960
H	-4.6504309	2.9182435	-3.6710960
H	1.8520426	-3.5830768	-4.7253318
H	3.5830768	1.8520426	-4.7253318
H	-1.8520426	3.5830768	-4.7253318
H	3.1230114	-0.8641049	-4.0535286
H	0.8641049	3.1230114	-4.0535286
H	-3.1230114	0.8641049	-4.0535286
H	1.8536556	-1.6963746	-3.1136436
H	1.6963746	1.8536556	-3.1136436
H	-1.8536556	1.6963746	-3.1136436
H	1.5135158	-1.1288854	-4.7672560
H	1.1288854	1.5135158	-4.7672560
H	-1.5135158	1.1288854	-4.7672560
H	5.3707211	-2.3444581	-5.8522020
H	2.3444581	5.3707211	-5.8522020
H	-5.3707211	2.3444581	-5.8522020
H	3.4368750	-3.8300419	-6.5402425
H	3.8300419	3.4368750	-6.5402425
H	-3.4368750	3.8300419	-6.5402425
H	2.4125606	-2.4207112	-6.7680433
H	2.4207112	2.4125606	-6.7680433
H	-2.4125606	2.4207112	-6.7680433
H	4.7367554	-1.9384643	-7.4528241
H	1.9384643	4.7367554	-7.4528241
H	-4.7367554	1.9384643	-7.4528241
H	4.3269074	-0.9056075	-6.0689698
H	0.9056075	4.3269074	-6.0689698
H	-4.3269074	0.9056075	-6.0689698

Coordinates of optimized structure of Λ_S (LS complex)

324

Energy =

Fe	6.4881810	1.0139436	-0.0382107
N	8.0880930	-0.1534529	-0.1218049
N	8.4863286	-0.3938173	-1.3957077
N	6.6676668	0.8448960	-1.9507926
N	4.8912232	2.1615261	-0.6760905
N	3.8342551	3.0655389	-2.4404618
N	0.5633042	5.3142908	0.6005290
N	1.7490192	4.5873071	2.3651701
N	-1.2287494	6.6102104	1.8742747

N	-2.9599968	7.9679567	1.3181476
N	-2.6102932	7.6602342	0.0444429
C	8.9809244	-0.7453807	0.6558007
H	8.8943565	-0.6936145	1.7412005
C	9.9788316	-1.3826443	-0.1145396
H	10.8448829	-1.9397310	0.2425027
C	9.6335474	-1.1347549	-1.4206642
H	10.1209352	-1.4233418	-2.3517335
C	7.7292380	0.1805716	-2.4119803
C	8.0419890	0.1251909	-3.7722844
H	8.9183075	-0.4126226	-4.1405075
C	7.2057936	0.8144533	-4.6422387
H	7.4220761	0.8101933	-5.7146858
C	6.1090463	1.5378364	-4.1571878
H	5.4879164	2.0894297	-4.8601184
C	5.8670770	1.5459645	-2.7828914
C	4.8477274	2.2635569	-2.0058143
C	3.1551128	3.4929268	-1.3123756
C	2.0212574	4.2942362	-1.1861791
H	1.4752632	4.6902954	-2.0430336
C	1.6177602	4.5658634	0.1239758
C	2.3631360	4.0703093	1.2369665
C	3.4742325	3.2378382	1.1107911
H	4.0189398	2.8403598	1.9677423
C	3.8563685	2.9365831	-0.1994098
C	0.6752320	5.3065324	1.9299522
C	-0.3175892	6.0607748	2.7065772
C	-0.3977200	6.2907693	4.0806454
H	0.3160582	5.8658106	4.7835222
C	-1.4180892	7.1188853	4.5649015
H	-1.4863818	7.3263536	5.6369450
C	-2.3286864	7.7059827	3.6946658
H	-3.1058021	8.3795559	4.0623536
C	-2.1827445	7.4218763	2.3347478
C	-3.4453442	8.3306512	-0.7336290
H	-3.3700146	8.2627615	-1.8189748
C	-4.3552224	9.0888920	0.0361615
H	-5.1489254	9.7447007	-0.3213569
C	-4.0141057	8.8362989	1.3424681
H	-4.4369715	9.2138270	2.2732703
C	2.3091838	4.5245155	3.7144749
H	2.2498997	5.5344235	4.1494569
H	3.3863149	4.3254856	3.5946672
C	1.6919388	3.4845354	4.6540271
H	0.5986531	3.6586236	4.6899201
C	2.2533788	3.7158141	6.0648449
H	2.0613126	4.7660515	6.3560475
H	3.3561871	3.6184753	6.0333524
C	1.6922367	2.7994398	7.1429631
H	0.5881421	2.8373300	7.1750681
H	2.0576141	3.1057930	8.1366872

H	1.9933610	1.7474639	6.9986441
C	1.9139130	2.0642504	4.1456224
H	1.5387530	1.9315178	3.1148953
H	1.3918848	1.3322871	4.7831926
H	2.9899179	1.8066144	4.1588796
Fe	-1.0139436	6.4881810	-0.0382107
N	0.1534529	8.0880930	-0.1218049
N	0.3938173	8.4863286	-1.3957077
N	-0.8448960	6.6676668	-1.9507926
N	-2.1615261	4.8912232	-0.6760905
N	-3.0655389	3.8342551	-2.4404618
N	-5.3142908	0.5633042	0.6005290
N	-4.5873071	1.7490192	2.3651701
N	-6.6102104	-1.2287494	1.8742747
N	-7.9679567	-2.9599968	1.3181476
N	-7.6602342	-2.6102932	0.0444429
C	0.7453807	8.9809244	0.6558007
H	0.6936145	8.8943565	1.7412005
C	1.3826443	9.9788316	-0.1145396
H	1.9397310	10.8448829	0.2425027
C	1.1347549	9.6335474	-1.4206642
H	1.4233418	10.1209352	-2.3517335
C	-0.1805716	7.7292380	-2.4119803
C	-0.1251909	8.0419890	-3.7722844
H	0.4126226	8.9183075	-4.1405075
C	-0.8144533	7.2057936	-4.6422387
H	-0.8101933	7.4220761	-5.7146858
C	-1.5378364	6.1090463	-4.1571878
H	-2.0894297	5.4879164	-4.8601184
C	-1.5459645	5.8670770	-2.7828914
C	-2.2635569	4.8477274	-2.0058143
C	-3.4929268	3.1551128	-1.3123756
C	-4.2942362	2.0212574	-1.1861791
H	-4.6902954	1.4752632	-2.0430336
C	-4.5658634	1.6177602	0.1239758
C	-4.0703093	2.3631360	1.2369665
C	-3.2378382	3.4742325	1.1107911
H	-2.8403598	4.0189398	1.9677423
C	-2.9365831	3.8563685	-0.1994098
C	-5.3065324	0.6752320	1.9299522
C	-6.0607748	-0.3175892	2.7065772
C	-6.2907693	-0.3977200	4.0806454
H	-5.8658106	0.3160582	4.7835222
C	-7.1188853	-1.4180892	4.5649015
H	-7.3263536	-1.4863818	5.6369450
C	-7.7059827	-2.3286864	3.6946658
H	-8.3795559	-3.1058021	4.0623536
C	-7.4218763	-2.1827445	2.3347478
C	-8.3306512	-3.4453442	-0.7336290
H	-8.2627615	-3.3700146	-1.8189748
C	-9.0888920	-4.3552224	0.0361615

H	-9.7447007	-5.1489254	-0.3213569
C	-8.8362989	-4.0141057	1.3424681
H	-9.2138270	-4.4369715	2.2732703
C	-4.5245155	2.3091838	3.7144749
H	-5.5344235	2.2498997	4.1494569
H	-4.3254856	3.3863149	3.5946672
C	-3.4845354	1.6919388	4.6540271
H	-3.6586236	0.5986531	4.6899201
C	-3.7158141	2.2533788	6.0648449
H	-4.7660515	2.0613126	6.3560475
H	-3.6184753	3.3561871	6.0333524
C	-2.7994398	1.6922367	7.1429631
H	-2.8373300	0.5881421	7.1750681
H	-3.1057930	2.0576141	8.1366872
H	-1.7474639	1.9933610	6.9986441
C	-2.0642504	1.9139130	4.1456224
H	-1.9315178	1.5387530	3.1148953
H	-1.3322871	1.3918848	4.7831926
H	-1.8066144	2.9899179	4.1588796
Fe	1.0139436	-6.4881810	-0.0382107
N	-0.1534529	-8.0880930	-0.1218049
N	-0.3938173	-8.4863286	-1.3957077
N	0.8448960	-6.6676668	-1.9507926
N	2.1615261	-4.8912232	-0.6760905
N	3.0655389	-3.8342551	-2.4404618
N	5.3142908	-0.5633042	0.6005290
N	4.5873071	-1.7490192	2.3651701
N	6.6102104	1.2287494	1.8742747
N	7.9679567	2.9599968	1.3181476
N	7.6602342	2.6102932	0.0444429
C	-0.7453807	-8.9809244	0.6558007
H	-0.6936145	-8.8943565	1.7412005
C	-1.3826443	-9.9788316	-0.1145396
C	-1.1347549	-9.6335474	-1.4206642
H	-1.4233418	-10.1209352	-2.3517335
C	0.1805716	-7.7292380	-2.4119803
C	0.1251909	-8.0419890	-3.7722844
H	-0.4126226	-8.9183075	-4.1405075
C	0.8144533	-7.2057936	-4.6422387
H	0.8101933	-7.4220761	-5.7146858
C	1.5378364	-6.1090463	-4.1571878
H	2.0894297	-5.4879164	-4.8601184
C	1.5459645	-5.8670770	-2.7828914
C	2.2635569	-4.8477274	-2.0058143
C	3.4929268	-3.1551128	-1.3123756
C	4.2942362	-2.0212574	-1.1861791
H	4.6902954	-1.4752632	-2.0430336
C	4.5658634	-1.6177602	0.1239758
C	4.0703093	-2.3631360	1.2369665
C	3.2378382	-3.4742325	1.1107911
H	2.8403598	-4.0189398	1.9677423

C	2.9365831	-3.8563685	-0.1994098
C	5.3065324	-0.6752320	1.9299522
C	6.0607748	0.3175892	2.7065772
C	6.2907693	0.3977200	4.0806454
H	5.8658106	-0.3160582	4.7835222
C	7.1188853	1.4180892	4.5649015
H	7.3263536	1.4863818	5.6369450
C	7.7059827	2.3286864	3.6946658
H	8.3795559	3.1058021	4.0623536
C	7.4218763	2.1827445	2.3347478
C	8.3306512	3.4453442	-0.7336290
H	8.2627615	3.3700146	-1.8189748
C	9.0888920	4.3552224	0.0361615
H	9.7447007	5.1489254	-0.3213569
C	8.8362989	4.0141057	1.3424681
H	9.2138270	4.4369715	2.2732703
C	4.5245155	-2.3091838	3.7144749
H	5.5344235	-2.2498997	4.1494569
H	4.3254856	-3.3863149	3.5946672
C	3.4845354	-1.6919388	4.6540271
H	3.6586236	-0.5986531	4.6899201
C	3.7158141	-2.2533788	6.0648449
H	4.7660515	-2.0613126	6.3560475
H	3.6184753	-3.3561871	6.0333524
C	2.7994398	-1.6922367	7.1429631
H	2.8373300	-0.5881421	7.1750681
H	3.1057930	-2.0576141	8.1366872
H	1.7474639	-1.9933610	6.9986441
C	2.0642504	-1.9139130	4.1456224
H	1.9315178	-1.5387530	3.1148953
H	1.3322871	-1.3918848	4.7831926
H	1.8066144	-2.9899179	4.1588796
Fe	-6.4881810	-1.0139436	-0.0382107
N	-8.0880930	0.1534529	-0.1218049
N	-8.4863286	0.3938173	-1.3957077
N	-6.6676668	-0.8448960	-1.9507926
N	-4.8912232	-2.1615261	-0.6760905
N	-3.8342551	-3.0655389	-2.4404618
N	-0.5633042	-5.3142908	0.6005290
N	-1.7490192	-4.5873071	2.3651701
N	1.2287494	-6.6102104	1.8742747
N	2.9599968	-7.9679567	1.3181476
N	2.6102932	-7.6602342	0.0444429
C	-8.9809244	0.7453807	0.6558007
H	-8.8943565	0.6936145	1.7412005
C	-9.9788316	1.3826443	-0.1145396
H	-10.8448829	1.9397310	0.2425027
C	-9.6335474	1.1347549	-1.4206642
H	-10.1209352	1.4233418	-2.3517335
C	-7.7292380	-0.1805716	-2.4119803
C	-8.0419890	-0.1251909	-3.7722844

H	-8.9183075	0.4126226	-4.1405075
C	-7.2057936	-0.8144533	-4.6422387
H	-7.4220761	-0.8101933	-5.7146858
C	-6.1090463	-1.5378364	-4.1571878
H	-5.4879164	-2.0894297	-4.8601184
C	-5.8670770	-1.5459645	-2.7828914
C	-4.8477274	-2.2635569	-2.0058143
C	-3.1551128	-3.4929268	-1.3123756
C	-2.0212574	-4.2942362	-1.1861791
H	-1.4752632	-4.6902954	-2.0430336
C	-1.6177602	-4.5658634	0.1239758
C	-2.3631360	-4.0703093	1.2369665
C	-3.4742325	-3.2378382	1.1107911
H	-4.0189398	-2.8403598	1.9677423
C	-3.8563685	-2.9365831	-0.1994098
C	-0.6752320	-5.3065324	1.9299522
C	0.3175892	-6.0607748	2.7065772
C	0.3977200	-6.2907693	4.0806454
H	-0.3160582	-5.8658106	4.7835222
C	1.4180892	-7.1188853	4.5649015
H	1.4863818	-7.3263536	5.6369450
C	2.3286864	-7.7059827	3.6946658
H	3.1058021	-8.3795559	4.0623536
C	2.1827445	-7.4218763	2.3347478
C	3.4453442	-8.3306512	-0.7336290
H	3.3700146	-8.2627615	-1.8189748
C	4.3552224	-9.0888920	0.0361615
H	5.1489254	-9.7447007	-0.3213569
C	4.0141057	-8.8362989	1.3424681
H	4.4369715	-9.2138270	2.2732703
C	-2.3091838	-4.5245155	3.7144749
H	-2.2498997	-5.5344235	4.1494569
H	-3.3863149	-4.3254856	3.5946672
C	-1.6919388	-3.4845354	4.6540271
H	-0.5986531	-3.6586236	4.6899201
C	-2.2533788	-3.7158141	6.0648449
H	-2.0613126	-4.7660515	6.3560475
H	-3.3561871	-3.6184753	6.0333524
C	-1.6922367	-2.7994398	7.1429631
H	-0.5881421	-2.8373300	7.1750681
H	-2.0576141	-3.1057930	8.1366872
H	-1.9933610	-1.7474639	6.9986441
C	-1.9139130	-2.0642504	4.1456224
H	-1.5387530	-1.9315178	3.1148953
H	-1.3918848	-1.3322871	4.7831926
H	-2.9899179	-1.8066144	4.1588796
H	-1.9397310	-10.8448829	0.2425027
C	-3.5877737	-3.5628470	-3.7934313
C	-2.7924353	-2.6221158	-4.7041030
C	-2.8499115	-3.1104007	-6.1623948
C	-2.2506448	-4.4837008	-6.4447722

C	-1.3678302	-2.4124960	-4.1998387
H	-4.5612238	-3.8196138	-4.2405925
H	-3.0607272	-4.5221492	-3.6770401
H	-3.2931327	-1.6356035	-4.6785104
H	-0.8016910	-3.3608813	-4.1818601
H	-1.3498090	-1.9850909	-3.1811562
H	-0.8251658	-1.7171622	-4.8630797
H	-2.7782789	-5.2947401	-5.9117651
H	-3.9041914	-3.1071843	-6.4994854
H	-2.3422234	-2.3561933	-6.7900570
H	-2.3270590	-4.7142919	-7.5201553
H	-1.1794118	-4.5338921	-6.1795830
C	3.5628470	-3.5877737	-3.7934313
C	3.5877737	3.5628470	-3.7934313
C	-3.5628470	3.5877737	-3.7934313
C	2.6221158	-2.7924353	-4.7041030
C	2.7924353	2.6221158	-4.7041030
C	-2.6221158	2.7924353	-4.7041030
C	3.1104007	-2.8499115	-6.1623948
C	2.8499115	3.1104007	-6.1623948
C	-3.1104007	2.8499115	-6.1623948
C	4.4837008	-2.2506448	-6.4447722
C	2.2506448	4.4837008	-6.4447722
C	-4.4837008	2.2506448	-6.4447722
C	2.4124960	-1.3678302	-4.1998387
C	1.3678302	2.4124960	-4.1998387
C	-2.4124960	1.3678302	-4.1998387
H	3.8196138	-4.5612238	-4.2405925
H	4.5612238	3.8196138	-4.2405925
H	-3.8196138	4.5612238	-4.2405925
H	4.5221492	-3.0607272	-3.6770401
H	3.0607272	4.5221492	-3.6770401
H	-4.5221492	3.0607272	-3.6770401
H	1.6356035	-3.2931327	-4.6785104
H	3.2931327	1.6356035	-4.6785104
H	-1.6356035	3.2931327	-4.6785104
H	3.3608813	-0.8016910	-4.1818601
H	0.8016910	3.3608813	-4.1818601
H	-3.3608813	0.8016910	-4.1818601
H	1.9850909	-1.3498090	-3.1811562
H	1.3498090	1.9850909	-3.1811562
H	-1.9850909	1.3498090	-3.1811562
H	1.7171622	-0.8251658	-4.8630797
H	0.8251658	1.7171622	-4.8630797
H	-1.7171622	0.8251658	-4.8630797
H	5.2947401	-2.7782789	-5.9117651
H	2.7782789	5.2947401	-5.9117651
H	-5.2947401	2.7782789	-5.9117651
H	3.1071843	-3.9041914	-6.4994854
H	3.9041914	3.1071843	-6.4994854
H	-3.1071843	3.9041914	-6.4994854

H	2.3561933	-2.3422234	-6.7900570
H	2.3422234	2.3561933	-6.7900570
H	-2.3561933	2.3422234	-6.7900570
H	4.7142919	-2.3270590	-7.5201553
H	2.3270590	4.7142919	-7.5201553
H	-4.7142919	2.3270590	-7.5201553
H	4.5338921	-1.1794118	-6.1795830
H	1.1794118	4.5338921	-6.1795830
H	-4.5338921	1.1794118	-6.1795830

8. Differences in ECD spectra between Λ_S and Λ_R complexes

In order to verify the circular dichroism and the helicity of the grid complexes, the ECD spectra of both diastereomers of the complex with Λ helicity are calculated. If the chirality of these molecules was mainly caused by the chirality of the ligands, the Λ_R and Λ_S complexes should exhibit ECD spectra which only differ by a sign of $\Delta\varepsilon$. TDDFT calculations on both diastereomers have been carried out using the PBE0 functional and a def2-SV(P) basis set for all atoms except iron for which a def2-TZVP was chosen as described in Section 7 of this SI. Only the Low-Spin complexes have been considered.

As shown in figure SI-9, both diastereomers exhibit ECD spectra which are virtually identical. This implies that the circular dichroism of these molecules is primarily caused by the helicity of the system and not the chiral sites of the ligands. Circular dichroism can thus be used in order to verify the helicity of these systems, confirming that the experimentally analyzed structures all belong to complexes with Λ helicity.

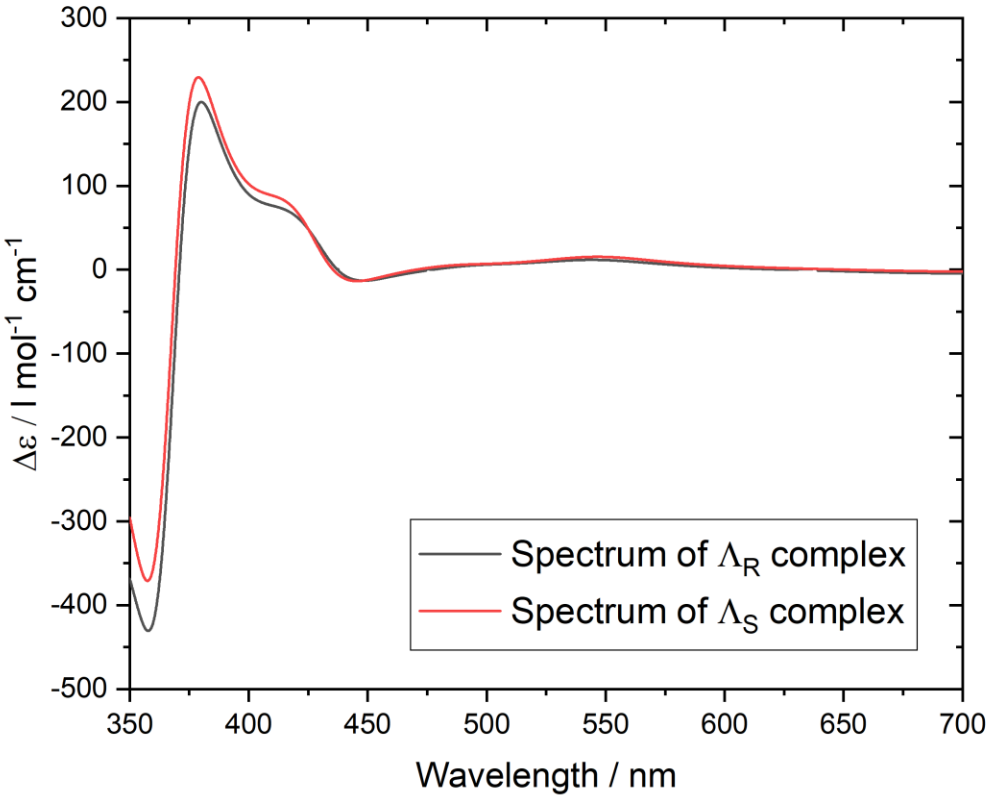

Figure SI-9: *ECD spectra of the Low-Spin Λ_R (black) and Λ_S (red) complexes as calculated using TDDFT (PBE0 functional). The diastereomers exhibit ECD spectra that are virtually identical, strongly implying that the circular dichroism of these systems is primarily caused by the helicity of the grid complex, not the chiral sites in the ligands.*

9. Magnetic measurements

All herein reported magnetic measurements were performed on an MPMS-XL7 and MPMS-XL5 magnetometers (Quantum Design). For standard magnetic as well as for photo-magnetic experiments, the temperature-dependent magnetization was recorded at B_{DC} = 0.1 T as an external magnetic field. The temperature sweeping rate was 3 K/min (standard measurements in the dark) or 0.3 K/min (photo-magnetic experiments) and it was the same for cooling and for heating modes. Each temperature data point was stabilized for 1 minute before the measurement. Gelatin capsule (standard measurements in the dark) was used as sample holders in the temperature range 1.8 ↔ 385 K. The very small diamagnetic contribution of the gelatin capsule was negligible to the overall magnetization, which was dominated by sample. The diamagnetic corrections of the molar magnetic susceptibilities were applied using Pascal's constants. The photo-magnetic measurements were performed by using a diode-pumped solid-state lasers (DPSS) Kvant (λ= 637 nm, 300 mW) coupled through an optical fiber to the cavity of an MPMS SQUID XL7 and the power on the sample surface was adjusted to 10 mW/cm^2. For

the photo-magnetic experiments, the small amount of sample (≈1 mg) was dispersed in melted eicosane, introduced into the plastic straw and congealed. The exact weight of samples was obtained by weighting and verified by comparison of thermal χT *vs.* T curve with a more accurately weighed sample of the same compound. After the cooling to 5 K, the sample was irradiated and the change in magnetization was followed. When the saturation point had been reached, the light was switched off, the temperature was increased at a rate of 0.3 K/min, and the magnetization was measured at 1 K intervals. We observed the most intense increase of magnetic moment under the red light irradiation (637 nm). T(LIESST) value was determined from the minimum of the $\partial(\chi T)/\partial T$ *vs.* T curve for the relaxation process.

10. Mössbauer spectroscopy

^{57}Fe Mössbauer Spectroscopy was performed in transmission geometry employing a constant acceleration spectrometer which was used in conjunction with a 512-channel analyzer in time-scale mode (WissEl GmbH). The 14.4 keV γ-radiation was delivered by a radioactive source containing ^{57}Co which was diffused in Rh. Source activity was 1.4 GBq. The calibration of the spectrometer was carried out with α-iron at room temperature (RT). Variable temperature measurements between 77 K and RT were conducted in a continuous flow cryostat (OptistatDN, Oxford Instruments). The spectral data were transferred from the multi-channel analyzer to a PC utilizing the public domain program Vinda running on an Excel 2003® platform. The analysis was performed with least-squares fits using Lorentzian line shapes which reveal the parameters isomer shift δ, the quadrupole splitting ΔE_Q and the line width at half maximum Γ.[9]

11. Chiral HPLC measurements

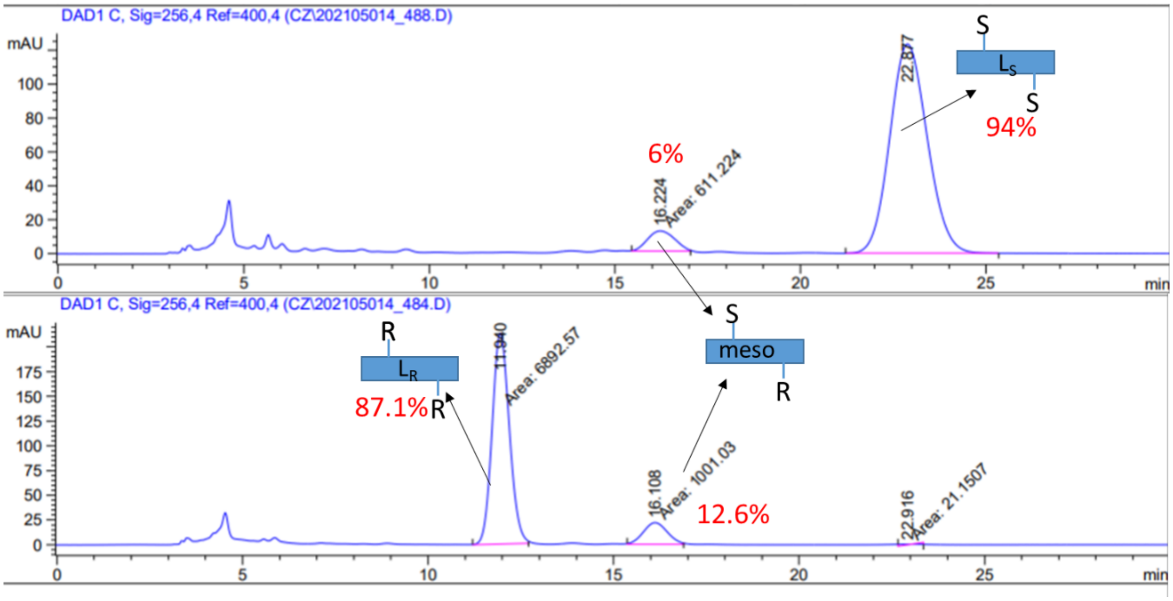

Figure SI-10: *Chromatograms of ligand L_S (top) and L_R (bottom) on chiral HPLC – conforming 94% purity of L_S and 87% purity from L_R. which is obtained from the ratios of the peak areas.*

12. References

[1] E. K. U. Gross, W. Kohn, *Adv. Quantum Chem.* **1990**, *21*, 255–291.

[2] R. Bauernschmitt, R. Ahlrichs, *Chem. Phys. Lett.* **1996**, *256*, 454–464.

[3] R. Bauernschmitt, R. Ahlrichs, *J. Chem. Phys.* **1996**, *104*, 9047–9052.

[4] R. Ahlrichs, M. Bär, M. Häser, H. Horn, C. Kölmel, *Chem. Phys. Lett.* **1989**, *162*, 165–169.

[5] J. P. Perdew, K. Burke, M. Ernzerhof, *Phys. Rev. Lett.* **1996**, *77*, 3865–3868.

[6] J. P. Perdew, M. Ernzerhof, K. Burke, *J. Chem. Phys.* **1996**, *105*, 9982–9985.

[7] F. Weigend, R. Ahlrichs, *Phys. Chem. Chem. Phys.* **2005**, *7*, 3297–3305.

[8] O. Treutler, R. Ahlrichs, *J. Chem. Phys.* **1995**, *102*, 346–354.

[9] H. P. Gunnlaugsson, *Hyperfine Interact.* **2016**, *237*, 79.
